# Influence of Plant Part Selection and Drying Technique: Exploration and Optimization of Antioxidant and Antibacterial Activities of New Guinea Impatiens Extracts

**DOI:** 10.3390/plants14071092

**Published:** 2025-04-01

**Authors:** Fabián Delgado Rodríguez, Gabriela Azofeifa, Silvia Quesada, Nien Tzu Weng Huang, Arlene Loría Gutiérrez, María Fernanda Morales Rojas

**Affiliations:** 1 Instituto de Investigaciones Farmacéuticas (INIFAR), Facultad de Farmacia, Universidad de Costa Rica, San José 11501-2060, Costa Rica; nien-tzu.weng@ucr.ac.cr (N.T.W.H.); arlene.loria@ucr.ac.cr (A.L.G.); maria.moralesrojas@ucr.ac.cr (M.F.M.R.); 2 Departamento de Bioquímica, Escuela de Medicina, Universidad de Costa Rica, San José 11501-2060, Costa Rica; gabriela.azofeifacordero@ucr.ac.cr (G.A.); silvia.quesada@ucr.ac.cr (S.Q.)

**Keywords:** *Impatiens hawkeri*, antioxidant, antibacterial, drying, HPTLC, polyphenols, correlation, principal component analysis

## Abstract

*Impatiens* L. plants are sources of polyphenols with antioxidant and antimicrobial activities. There are scarce data about these effects in the case of *Impatiens hawkeri* W. Bull, a relevant species in ornamental plant industry with ethnobotanical backgrounds. The aim of this study is to provide information regarding the antioxidant and the antibacterial properties of the ethanol extracts of *I. hawkeri* to support new applications. HPTLC was used to estimate the concentration of seven known bioactive metabolites reported among *Impatiens* plants. Total phenolics, flavonoids, and monomeric anthocyanins were also measured. An orthogonal platform with chemical and biological in vitro assays was used to evaluate the antioxidant activity of the extracts. Antibacterial activity was determined by broth microdilution assay on human pathogenic bacteria. The results were integrated by correlation and principal component analysis to identify the most promissory plant part and drying technique to optimize the evaluated activities. Data suggest the tentative identification of bioactive chemical markers for the antioxidant and antibacterial activities of the extracts (quercetin and rutin). Freeze-dried leaves and flowers are the most promissory parts of *I. hawkeri* for the development of antioxidant nutraceuticals or preservatives. The results demonstrate that phenolic compounds play a major role in the antioxidant and antibacterial activities of *I. hawkery* extracts.

## 1. Introduction

Medicinal herbs with antioxidant activity have been used for the development of nutraceuticals for the prevention of cancer, cardiovascular, and neurodegenerative diseases due to their free radical scavenging capability [1]. Among the compounds responsible for the antioxidant activity of these plants, polyphenols have notorious relevance [2,3]. Furthermore, medicinal plants have been considered as sources of extracts and natural products with antioxidant activity that can be used as an alternative to common synthetic preservatives that have limitations due to their toxicity and potential health risks [4,5]. This is especially important for those medicinal plants with the capability to inhibit the growth of foodborne pathogens and microorganisms implicated in food spoilage [4].

Drying of medicinal plants is a clue post-harvesting process to warrant their preservation and conservation of bioactive compounds. Moisture reduction promotes the inhibition of microbial growth, and reduction in enzymatic activity, and favors a delay in spoilage [6,7]. As drying lets a reduction in plant material volume and weight, it facilitates other processes such as transportation, storage, and availability in different seasons through the year. Nowadays, there are many drying techniques that can be used to reduce moisture in plant materials and extend their shelf-life. Nevertheless, different techniques offer distinct advantages and disadvantages related to the outcomes in terms of the quality of dried material and costs. Among common drying techniques are oven-drying, shade-drying, and freeze-drying. Oven-drying offers a more efficient alternative than drying in natural conditions (i.e., sun-drying and shade-drying) since it is possible to set relatively high thermal gradient between internal and external layers of material under drying. Despite the fact that heat application can facilitate the establishment of high thermal gradients and moisture diffusivity, it can also promote the thermal degradation of sensitive compounds [6].

In the case of shade-drying, it takes place on relatively low-temperature conditions; therefore, the preservation of thermal and volatile compounds is expected. Nonetheless, as the drying process is slow, plant material can keep its enzymatic activity for a longer time, giving place to the chemical transformation of bioactive compounds. This factor might counteract the expected benefit of drying under lower temperatures [6]. In freeze-drying, the process takes place under cold temperatures and strong vacuum. These conditions allow the preservation of temperature-sensitive compounds in plant material and a decline in the activity of its degrative enzymes. The major drawbacks of freeze-drying are related to the cost due to long drying times and the requirement of effective refrigeration and strong vacuum systems [6,8]. Although it is anticipated that freeze-drying promotes a higher preservation of thermal-sensitive components, the loss of highly volatile compounds responsible for the aroma of medicinal plants has been reported [9].

Despite the described trends for outcomes of the mentioned drying techniques, it is important to consider that the final quality of dried products also depends on the specific processed plant material. For instance, a particular medicinal plant can produce specific oxidative and hydrolytic enzymes that could influence the properties of dried material differently in comparison to other plants. In addition, the activity of these enzymes and their impact in dried material composition can also vary with drying conditions [10,11]. It is noteworthy that water content varies widely among different plant material (i.e., different species and different plant parts) being necessary to define appropriate conditions to promote an efficient drying process (i.e., drying temperature, drying time, air flux regulation, environmental or equipment humidity, among others). So, the most appropriate way to choose a suitable drying technique is to carry out drying studies; hence, stakeholders in medicinal plants’ quality can take decisions based on experimental data for each case. This suggests the requirement of tailored drying procedures for each plant material in order to optimize their quality.

On the other hand, *Impatiens* L. plants are known sources of important bioactive polyphenols, including naphthoquinones, coumarins, flavonoids, and their glycosidic derivates [12]. Some of them are used in the production of nutraceuticals, including quercetin, kaempferol, and quercetin 3-*O*-β-D-rutinoside (known as rutin) [13,14,15]. These compounds have been identified as antioxidant and antibacterial secondary metabolites [16,17,18]. Other polyphenols with antioxidant and antimicrobial activities have been reported among *Impatiens* plants, including scopoletin [12,19,20], 2-hydroxy-1,4-naphthoquinone (known as lawsone) [21,22], and 2-methoxy-1,4-naphtoquinone [21,23]. These compounds are not only relevant for their biological effects; for instance, lawsone has been used as a cosmetic pigment [24].

Due to the presence of bioactive phenolic metabolites, *Impatiens* plant extracts have shown relevant antioxidant and antibacterial activities [25,26,27]. They have been investigated as potential preservatives for fruits [28,29] and medicinal plant products [30]. In the case of 2-methoxy-1,4-naphtoquinone, it has been studied as an active principle for the development of antimicrobial pharmaceutical products among other applications [31]. In addition, derived plant naphthoquinones are interesting starting points for the synthesis of analogous compounds with enhanced antimicrobial activity [32].

*Impatiens hawkeri* W. Bull is an important species in the ornamental plant industry due to its characteristic flowers. The total annual sales of this plant in the United States are valued at USD 81,564,000 according to the 2019 Census of Horticultural Specialties of the United States Department of Agriculture [33]. Additionally, *I. hawkeri* has been used in Papua New Guinea as an edible plant and a traditional medicine to promote pregnancy and to treat stomachache in children, scabies, and parturition pain [34,35,36].

There are previous reports indicating differences in the phenolic constitution and magnitudes of antimicrobial and antioxidant activities among extracts prepared from different parts of *Impatiens* plants [26,37,38]; however, few have been investigated in the case of *I. hawkeri.* It has been reported that whole-plant extract from *I. hawkeri* can have antioxidant and antimicrobial activity [39]. Nevertheless, there is no available information regarding the relationships between specific compounds and the observed activities or differences in the chemical constitutions and biological activities among extracts prepared from different plant parts. Therefore, in the present work, the tentative determination of bioactive polyphenols with industrial and pharmacological relevance, previously reported in other *Impatiens* plants, has been carried out for the ethanol extracts obtained from different parts of *I. hawkeri*. The antioxidant activity of the extracts was evaluated using in vitro chemical and biological approaches. The antibacterial activity of the extracts was determined on human pathogenic bacteria. The effect of the drying technique applied on *I. hawkeri* leaves over the chemical features and bioactivities of their extracts was also investigated. The aim of this study is to provide evidence to support new applications for *I. hawkeri*, turning this species into something more than an ornamental plant, taking advantage of the current knowledge and cumulative experience in agricultural production systems for this plant.

## 2. Results

### 2.1. Quantitative Estimation of Bioactive Polyphenols by High-Performance Thin-Layer Chromatography (HPTLC)

**Figure 1:**
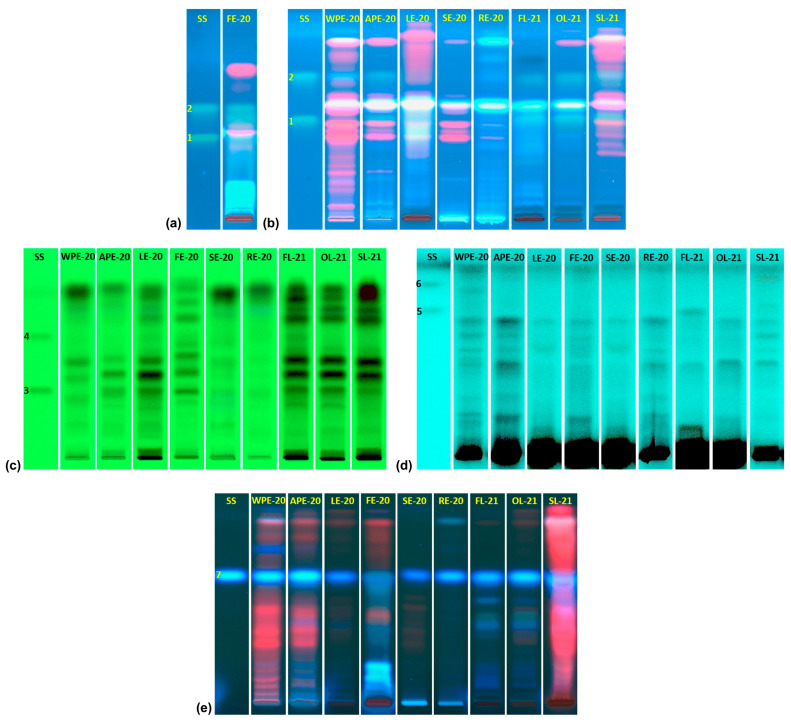
Chromatograms for the quantitative estimation of quercetin (1), kaempferol (2), rutin (3), isoquercetin (4), lawsone (5), 2-MNQ (6), and scopoletin (7). (**a**) Chromatograms observed under 366 nm UV light after development of the chromatographic plate with mobile phase 1 (toluene–ethyl acetate–formic acid (60:45:3 *v*/*v*/*v*)). (**b**) Chromatograms observed under 366 nm UV light after consecutive development of the chromatographic plate with the mobile phase 1 and mobile phase 2 (toluene–ethyl acetate–*n*-hexane–formic acid (60:30:10:3 *v*/*v*/*v*/*v*)). (**c**) Chromatograms observed under 254 nm UV light after development of the chromatographic plate with mobile phase 3 (butan-2-ol–*n*-butanol–ethyl acetate–formic acid (60:40:15:10 *v*/*v*/*v*/*v*)). (**d**) Chromatograms observed under 254 nm wavelength UV light after consecutive development with mobile phases 4 (toluene–ethyl acetate–acetic acid (80:30:3 *v*/*v*/*v*)) and 5 (toluene–ethyl acetate–*n*-hexane–acetic acid (80:30:20:10 *v*/*v*/*v*/*v*)). (**e**) Chromatograms observed under 366 nm wavelength UV light after consecutive development with mobile phases 6 (chloroform–ethyl acetate–formic acid (60:30:10 *v*/*v*/*v*)) and 7 (chloroform–ethyl acetate (60:40 *v*/*v*)). SS: standard solution (1: quercetin 350 ng/band; 2: kaempferol 300 ng/band; 3: rutin 850 ng/band; 4: isoquercetin 850 ng/band; 5: lawsone 330 ng/band; 6: 2-MNQ 640 ng/band; 7: scopoletin 240 ng/band); WPE-20: whole-plant extract from material collected in 2020; APE-20: aerial-part extract from material collected in 2020; LE-20: leaf extract from material collected in 2020; FE-20: flower extract from material collected in 2020; SE-20: stem extract from material collected in 2020; RE-20: root extract from material collected in 2020; FL-21: freeze-dried leaf extract from material collected in 2021; OL-21: oven-dried leaf extract from material collected in 2021; SL-21: shade-dried leaf extract from material collected in 2021.

Plant material was collected on two different dates. Material collected in February 2020 was used to evaluate the influence of the plant part on the examined properties of the extracts, whereas material collected in February 2021 was used to determine the effect of the drying technique in the same parameters of leaf extracts due to the availability of leaves and their best performance in most of the carried-out assays (see the results provided below). Figure 1 shows the obtained chromatograms for each extract using different mobile phase systems. Qualitative differences are observed among extracts from different plant parts. Interestingly, extract from shade-dried leaves shows a more complex composition in comparison to extracts prepared from leaves dried by oven-drying and freeze-drying.

Table 1 summarizes the results obtained for the quantitative estimation of the targeted bioactive polyphenols in the ethanol extracts from different plant parts of *I. hawkeri* based on the HPTLC–densitometric analysis of the chromatographic plates (sample densitograms are available in Figures S1–S5 in the Supplementary Material File). Scopoletin was detected in all the extracts. The highest concentration of this compound is present in the extract from oven-dried roots collected in February 2020 (RE-20). The highest quantity of rutin, a quercetin glycoside, has been determined in the extract from freeze-dried flowers collected in February 2020 (FE-20), whereas quercetin and kaempferol are at highest concentration in the extract from oven-dried aerial parts collected in February 2020 (APE-20). Isoquercetin, another quercetin glycoside, was only determined in the extract from oven-dried leaves collected in February 2020 (LE-20). The extract from oven-dried whole plants collected in February 2020 (WPE-20) has the second-highest amount of kaempferol, but it has a relatively low concentration of scopoletin in comparison to APE-20 and LE-20. In the case of the extract from oven-dried stems collected in February 2020 (SE-20), only low quantities of rutin and scopoletin were determined. Lawsone and 2-MNQ were not detected in any extract obtained from different plant parts collected in 2020.

Drying studies are useful to define methodologies that enhance bioactive compound concentration in plant extracts during their production. In the case of *I. hawkeri*, leaves are more abundant than flowers (see Table S1 in Supplementary Material File). Furthermore, fast discoloration and fermentation are observed in flowers during oven-drying and shade-drying, respectively. For these reasons and the antioxidant and the antimicrobial potential observed for the corresponding extract (see the results in sections below), leaves were selected to analyze the influence of drying technique on polyphenol concentration in extracts, even though LE-20 has the second highest level of SPC after FE-20. For this reason, a second collection of *I. hawkeri* leaves was carried out in February 2021, and the material was used to evaluate the effect of drying on the chemical and biological properties of the ethanol extracts produced from it. Table 2 demonstrates that the extract from shade-dried leaves collected in February 2021 (SL-21) has the highest concentrations of quercetin and kaempferol. The extract from oven-dried leaves collected in February 2021 (OL-21) has the highest levels of rutin and scopoletin, and the extract from freeze-dried leaves collected in February 2021 (FL-21) has the highest concentrations of lawsone and isoquercetin. Compound 2-MNQ was not detected in any extract produced from leaves collected in 2021.

### 2.2. Determination of TPC, TFC, and TMA Contents

HPTLC analyses provide information about the concentration of targeted bioactive compounds. However, there is the possibility that other phenolic compounds contribute to the evaluated activities and were omitted by HPTLC analyses. For this reason, it is relevant to quantify the total amount of polyphenols, flavonoids, and anthocyanins. Table 3 shows that LE-20 has the highest values for total polyphenols and flavonoids among extracts prepared with material collected in 2020. In the case of monomeric anthocyanins, they were only detectable in FE-20 and LE-20.

On the other hand, regarding the effect of drying conditions, data in Table 4 demonstrate that freeze-drying allows the production of the leaf extract with the best profile for TPC, TFC, and TMA values followed by oven-drying. Shade-drying significantly reduces the yield of these contents in the leaf extract. This technique demonstrated the worst performance for TPC and TFC yields.

### 2.3. Determination of In Vitro Antioxidant Activity Using Chemical Assays

Table 5 summarizes the results obtained for the extracts prepared from different plant parts. The results from oxygen radical absorbance capacity assay (ORAC) indicate that FE-20 is the most active extract, while data from ferric reducing antioxidant power assay (FRAP) demonstrate that LE-20 presents the best reducing antioxidant power. According to the DPPH radical scavenging activity assay, LE-20 and FE-20 are the most active extracts. Data from ferrous iron chelating activity assay (FICA) demonstrate that LE-20 has the highest chelating activity against iron (II) ion. The results show that LE-20 extract tends to be the most active in the different chemical antioxidant activity assays except for ORAC.

Table 6 summarizes the chemical antioxidant activity of *I. hawkeri* extracts obtained from leaves dried by different techniques. Data indicate that freeze-drying optimizes the antioxidant activity according to ORAC, DPPH, and FRAP assays of *I. hawkeri* leaf extracts, whereas shade-drying promotes an optimization of the chelating activity against the iron (II) ion.

### 2.4. Determination of In Vitro Antioxidant Activity Using Biological Assays

**Figure 2:**
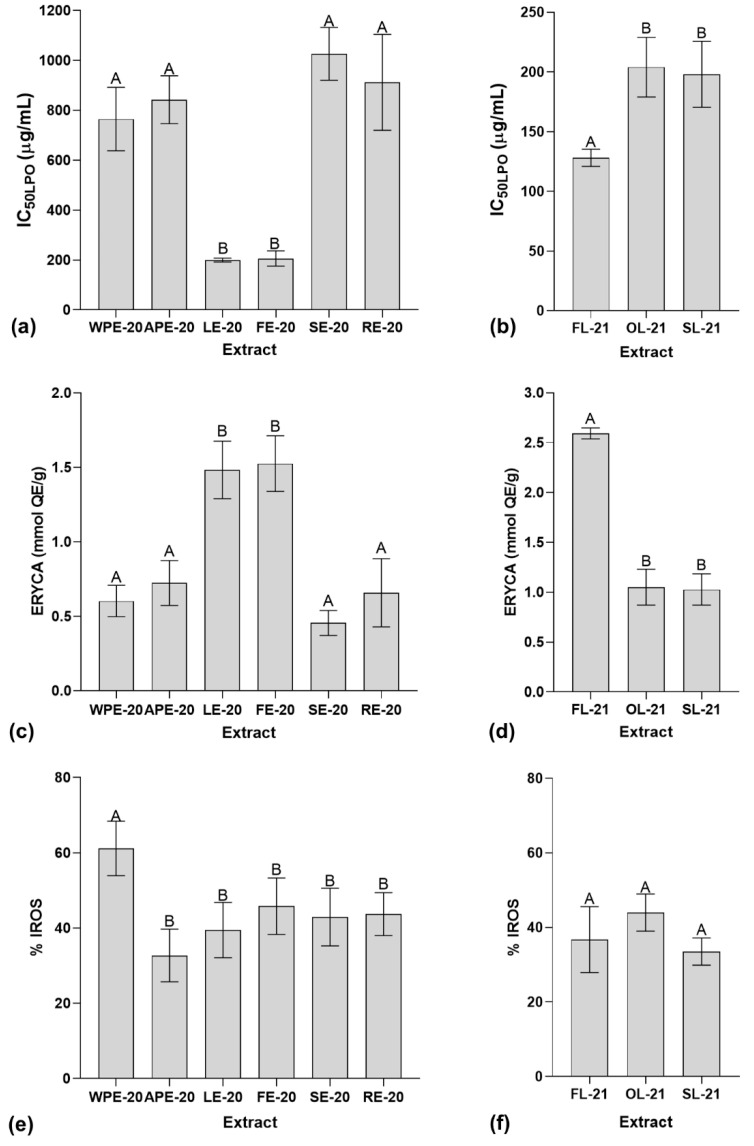
Antioxidant activity of the extracts determined by in vitro biological assays. (**a**) LPO assay results for extracts from different parts of *Impatiens hawkeri.* (**b**) LPO assay results for extracts obtained from *Impatiens hawkeri* leaves dried by different techniques. (**c**) ERYCA assay results for extracts from different parts of *Impatiens hawkeri.* (**d**) ERYCA assay results for extracts obtained from *Impatiens hawkeri* leaves dried by different techniques. (**e**) IROS assay results for extracts from different parts of *Impatiens hawkeri.* (**f**) IROS assay results for extracts obtained from *Impatiens hawkeri* leaves dried by different techniques. Error bars indicate standard deviation. Different letters above each bar indicate significant differences (*p* < 0.05). LPO: lipid peroxidation inhibition assay; ERYCA: erythrocyte cellular antioxidant activity assay; IROS: intracellular reactive oxygen species inhibition assay; IC_50LPO_: half-maximal inhibitory concentration against rat liver homogenate lipid peroxidation; QE: quercetin equivalents; WPE-20: whole-plant extract from material collected in 2020; APE-20: aerial-part extract from material collected in 2020; LE-20: leaf extract from material collected in 2020; FE-20: flower extract from material collected in 2020; SE-20: stem extract from material collected in 2020; RE-20: root extracts from material collected in 2020; FL-21: freeze-dried leaf extract from material collected in 2021; OL-21: oven-dried leaf extract from material collected in 2021; SL-21: shade-dried leaf extract from material collected in 2021.

The results from the lipid peroxidation inhibition assay (LPO) for the extracts from different plant parts of *I. hawkeri* are indicated in Figure 2a. They demonstrate that LE-20 and FE-20 extracts have the most potent inhibitory activity against rat liver homogenate lipid peroxidation with IC_50LPO_ values of 199.54 ± 7.68 µg/mL and 206.13 ± 30.31 µg/mL, respectively. In the case of extracts produced from leaves dried by different techniques, Figure 2b shows that FL-21 is the most active extract with a IC_50LPO_ of 128.07 ± 7.20 µg/mL. All extracts were significantly different to quercetin used as positive control (IC_50LPO_ = 4.67 ± 0.71 µg/mL; *p* < 0.05).

The erythrocyte cellular antioxidant activity assay (ERYCA) results showed in Figure 2c,d demonstrate that LE-20 and FE-20 are the extracts from different parts of *I. hawkeri* with the best antioxidant activity against hemolysis of erythrocytes exposed to oxidative stress (ERYCA values of 1.48 ± 0.19 mmol QE/g and 1.53 ± 0.19 mmol QE/g, respectively). Furthermore, in the case of extracts produced from *I. hawkeri* leaves dried by different techniques, FL-21 is the most active extract (ERYCA value of 2.59 ± 0.05 mmol QE/g).

Data provided in Figure 2e,f show that WPE-20 (the test concentration of 100 µg/mL) is the most potent antioxidant extract in the intracellular reactive oxygen species inhibition assay (IROS) when compared to other extracts obtained from different parts of *I. hawkeri* (% IROS = 61.16 ± 7.26%). It was the only one without a significant difference in comparison to quercetin (the test concentration of 25 µg/mL) used as positive control (% IROS = 53.31 ± 5.6%; *p* > 0.05). On the other hand, this assay did not show significant differences among extracts produced from *I. hawkeri* leaves dried by different techniques.

### 2.5. In Vitro Antibacterial Microdilution Assay

According to Table 7, among extracts obtained from different plant parts, only LE-20 and FE-20 showed antibacterial activity under the broth microdilution assay used for the determination of the minimal inhibitory concentration (MIC) of the extracts. Only Gram-positive bacteria showed susceptibility to the extracts.

In the case of the antibacterial activity of the extracts obtained from *I. hawkeri* leaves dried by different techniques (Table 8), OL-21 showed the best activity profile. However, again, the antibacterial activity of the leaf extracts was restricted to Gram-positive bacteria.

### 2.6. Correlation and Principal Component Analysis (PCA)

**Figure 3:**
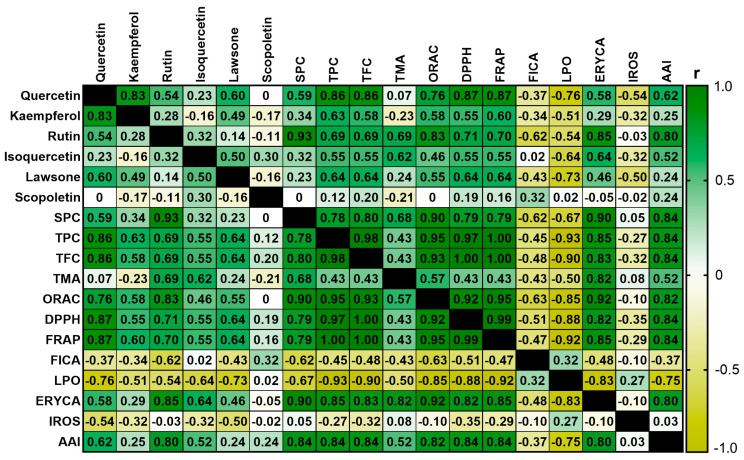
Spearman’s rank correlation matrix for polyphenolic characterization parameters and data from antioxidant and antibacterial activity assays. SPC: sum of polyphenols determined by HPTLC; TPC: total phenolic content; TFC: total flavonoid content; TMA: total monomeric anthocyanins content; ORAC: results from oxygen radical absorbance capacity assay; DPPH; results from 2,2-diphenyl-1-picrylhydrazylradical (DPPH) radical scavenging activity assay; FRAP: results from ferric reducing antioxidant power assay; FICA: results from ferreous iron chelating activity assay; LPO: results from lipid peroxidation inhibition assay; ERYCA: results from erythrocyte cellular antioxidant activity assay; IROS: results from intracellular reactive oxygen species inhibition assay; AAI: antibacterial activity index; r: Spearman’s rank correlation coefficient.

Figure 3 shows the Sperman’s rank correlation matrix for analyzed parameters on the extracts. Regarding significant correlations (*p* < 0.05) between the content of polyphenols defined by HPTLC chromatography and the magnitude of the antioxidant and antibacterial activities, the content of quercetin correlates with the results of the in vitro chemical antioxidant activity assays, except for the FICA assay. In addition, the quercetin content also presents a significant correlation with IC_50LPO_ values. The rutin content correlates with the results from the in vitro chemical antioxidant activity assays except for the FICA results. The content of this metabolite is positively correlated with the ERYCA values and the antibacterial activity index (AAI). Lawsone concentration is significantly correlated with LPO assay data; however, it has neither a significant correlation with in vitro chemical antioxidant activity assays nor the AAI results. The content of scopoletin, kaempferol, and isoquercetin did not show significant correlations with any of the variables determined by in vitro antioxidant activity assays or AAI values. The SPC variable has the same significant correlations described for rutin.

Additionally, TPC and TFC variables have significant correlations with all the parameters defined through the in vitro antioxidant and antibacterial activity assays, except for FICA and IROS results. TMA shows a positive and significant correlation with the results of ERYCA. Data from in vitro chemical antioxidant activity assays correlate significantly with the results from ERYCA and LPO biological assays, except for FICA results.

**Figure 4:**
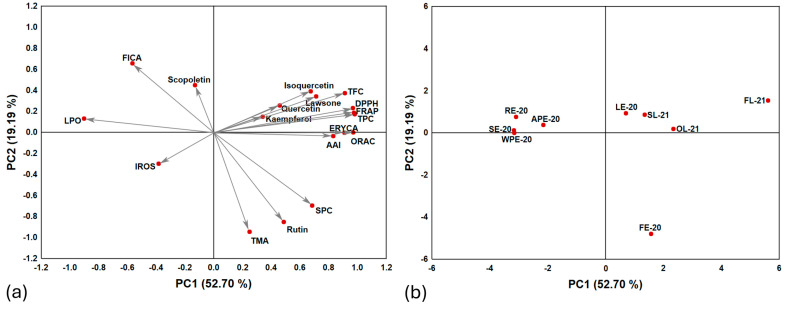
Loading plot (**a**) and score plot (**b**) from the principal component analysis (PCA) applied to the results obtained by *I. hawkeri* extracts analysis. PC1: first principal component; PC2: second principal component; SPC: sum of polyphenols determined by HPTLC; TPC: total phenolic content; TFC: total flavonoid content; TMA: total monomeric anthocyanins content; ORAC: results from oxygen radical absorbance capacity assay; DPPH: results from 2,2-diphenyl-1-picrylhydrazyl radical (DPPH) radical scavenging activity assay; FRAP: results from ferric reducing antioxidant power assay; FICA: results from ferreous iron chelating activity assay; LPO: results from lipid peroxidation inhibition assay; ERYCA: results from erythrocyte cellular antioxidant activity assay; IROS: results from intracellular reactive oxygen species inhibition assay; AAI: antibacterial activity index; WPE-20: whole-plant extract from material collected in 2020; APE-20: aerial-part extract from material collected in 2020; LE-20: leaf extract from material collected in 2020; FE-20: flower extract from material collected in 2020; SE-20: stem extract from material collected in 2020; RE-20: root extract from material collected in 2020; FL-21: freeze-dried leaf extract from material collected in 2021; OL-21: oven-dried leaf extract from material collected in 2021; SL-21: shade-dried leaf extract from material collected in 2021.

According to the principal component analysis (PCA) (Figure 4), it is possible to reduce the original 18 variables (dimensions) to only two, first principal component (PC1) and second principal component (PC2). These alternative variables can be summarized in a two-dimensional system that represents 71.89% of data total variance. PC1 and PC2 contribute with 52.70% and 19.19%, respectively. Regarding the existence of relationships between chemical features and biological activities observed in the loading plot (Figure 4a), based on the closeness between the points of each variable, it is possible to observe that TPC, TFC, DPPH, AAI, ORAC, FRAP, and ERYCA have a high positive correlation with each other. As expected, these variables show a negative correlation with LPO (vectors toward opposite directions) consistently with the inversely proportional relationship between the corresponding IC_50LPO_ values and the magnitude of the antioxidant activity. Furthermore, lawsone, kaempferol, quercetin, and isoquercetin tend towards a positive correlation with TPC, TFC, DPPH, AAI, ORAC, FRAP, and ERYCA. For the IROS percentage and scopoletin content, the angles and the small magnitude of their vectors in the loading plots, and their separation to other parameters included in PCA, indicate low correlations, especially with those variables determined by antioxidant and antibacterial activity assays.

The score plot (Figure 4b) demonstrates that the extracts from flowers and leaves are in the positive-values zone of PC1, whereas the extracts for the remaining plant parts are in the negative-values zone. There are two major groups of extracts in the score plot: the first one is integrated by extracts derived from oven-dried and shade dried leaves (LE-20, SL-21, and OL-21), and the second group is composed by extracts from plant parts other than flowers and leaves. There are two isolated extracts corresponding to FE-20 and FL-21. In the case of FE-20, its position in the score plot is determined by its relatively high TMA, SPC, and rutin contents. The position of FL-21 is mainly determined by its higher antioxidant activity, TPC, and TFC levels.

## 3. Discussion

### 3.1. Quantitative Estimation of Bioactive Polyphenols by High-Performance Thin-Layer Chromatography (HPTLC)

Observed qualitative differences in the chromatograms might be explained by chemical variability of the composition from each plant part. Chemical changes that can take place under different thermal conditions, including oxidation, hydrolysis, and enzymatic reactions, are additional factors that can contribute to the observed differences [10,40,41]. Freeze-drying tends to conserve thermal-sensitive compounds and protects them from oxidation by degradative enzymes present in the plant material [10]. The preservation of aroma, taste, and color is another advantage reported for freeze-drying; however, this technique implies a high cost of operation due to the price of the equipment and energy consumption [8]. In the case of oven-drying, it is a traditional drying method in industrial facilities. High-temperature techniques protect material from degradative enzymes by heat inactivation [10]; nevertheless, high temperatures can degrade thermolabile compounds. Sometimes this can be desirable; for instance, lignin or glycoside degradation induced by heat application can release bioactive polyphenols from plant material [42,43]. Shade-drying is a conventional method for drying plant material at an industrial scale due to their low cost in terms of energy and simple operations. Even though shade-drying offers protection to light sensitive compounds, it does not protect bioactive compounds from degradative enzymes present in plant material [10].

Variations found in the quantitative distribution of polyphenols among *I. hawkeri* plant parts are consistent with previously reported results for other species of *Impatiens* genus and medicinal plants such as *Hypericum perforatum* L. and *Ginkgo biloba* L. [37,38,44,45,46]. The relevance of these findings is the identification of plant parts that can be useful to produce extracts with high concentrations of bioactive components. The yield of compound extraction can be also influenced by the drying technique applied to plant material. Several factors influence the quantity and quality of extracted substances, including the thermal instability of compounds, volatility, hydrolysis, and the action of enzymes present in plant material (e.g., glycosylases, oxidases, and peroxidases) that can be activated or inactivated during heat application [8,9,10,11,43]. Each factor may have a different impact on each compound yield. These factors might be a reasonable explanation for the variability observed among determined polyphenols by HPTLC assays for *I. hawkery* leaf extracts prepared with material collected in February 2021.

Data in Table 2 show that oven-drying (OL-21) allows higher yield for rutin, followed by freeze-drying (FL-21) and shade-drying (SL-21). Isoquercetin is only detectable in FL-21. In the case of flavonoid aglycones, quercetin and kaempferol yields are higher with shade-drying followed by oven-drying and freeze-drying. We hypothesize that this trend might be explained by a combination of factors related to drying techniques and plant material. Higher levels of quercetin in SL-21 can be attributed to a higher influence of hydrolysis of quercetin glycosides during shade-drying process due to plant enzymatic activity. Despite the fact that hydrolysis can take place in oven-drying due to the application of high temperatures, these conditions can also reduce the influence of enzymatic hydrolysis in plant material; therefore, the quercetin yield in oven-drying can be influenced by two opposite factors, a reduction in the activity of hydrolytic enzymes in plant material and thermal-induced hydrolysis, and the balance between these phenomena can explain the lower quantity of quercetin in OL-21 in comparison to SL-21. In the case of freeze-drying, low temperature can reduce the enzymatic activity in plant material and provides protection to glycosidic compounds against thermal degradation; as a result, lower quantities of quercetin aglycone are released, resulting in a lower concentration of this compound in FL-21. It is remarkable that kaempferol concentrations follow a similar trend than quercetin levels; this observation suggests that the yield of both flavonoids aglycones is governed by the same factors.

The order for rutin yields observed in Table 2 can be also explained by a similar hypothesis. The degradation of rutin can take place during shade-drying due to enzymatic activity; therefore, a higher extent of the hydrolysis of quercetin glycosides provides a potential explanation for the lower levels of rutin in SL-21. In the case of oven-drying, balance between the inactivation of enzymatic activity in plant material and thermal mediated hydrolysis can result in higher yields for rutin in OL-21 in contrast to SL-21. Interestingly, rutin quantity in FL-21 is lower than OL-21; however, it is unknown whether rutin is derived from more complex glycosides that can be hydrolyzed with oven-drying giving rutin in higher levels than freeze-drying. Lower levels of glycosidic polyphenols in freeze-dried plants in contrast to material dried by other techniques, such as air-drying and vacuum-drying, have been previously reported [9]; therefore, it is necessary to analyze the implicated mechanism in further research to provide a comprehensive explanation of these phenomena. Another relevant observation related to results is that isoquercetin is only present in FL-21; this result can be explained by the protective effect of freeze-drying against the enzymatic and thermal degradation of glycosidic phenolics due to low-temperature conditions.

Regarding the selection of the determined compounds over others reported among *Impatiens* plants for their HPTLC analysis, their relevance is based on their bioactivity profile. As explained in the introduction, selected compounds are recognized by their antioxidant or antimicrobial activities [16,17,18,19,20,21,22,23]. In fact, compounds such as quercetin, kaempferol, and rutin are major components of nutraceutical products [13,14,15,16,17,18]. Hence, the determination of these bioactive polyphenols can be useful as evidence to support the development of healthcare products from *I. hawkeri*.

Furthermore, targeted compounds are known for their capability to exert other biological effects; therefore, their identification can give foundation to further research dealing with the evaluation of more biological activities. These investigations can extend the potential applications for *I. hawkeri.* For instance, quercetin is known by their anti-inflammatory, immunomodulatory, and cardioprotective effects [47]. Kaempferol is recognized by its anti-inflammatory, anti-diabetic, and protective properties on vascular, renal, and gastrointestinal systems [48]. Rutin also has a wide range of reported biological activities including analgesic, anti-inflammatory, antidepressant, anticonvulsant, antihypertensive, anti-ulcerogenic, and anti-platelet aggregation properties [16]. Isoquercetin has gained attention due to its antiviral activity; however, it has demonstrated additional biological effects including anti-inflammatory, anticoagulant, and immunomodulatory activities [49,50]. In the case of lawsone, in addition to being a relevant pigment for cosmetics, this compound has anti-inflammatory properties; nonetheless, it is important to ensure appropriate levels for this substance in order to avoid potential toxicological effects related to its cytotoxicity [24,51]. Compound 2-MNQ has demonstrated neuroprotective properties on in vitro experiments [23]. Scopoletin is another relevant bioactive compound characterized by its anti-inflammatory, hepatoprotective, antidiabetic, and vascular protective effects [52]. It is relevant to highlight that quercetin, kaempferol, rutin, lawsone, and 2-MNQ are compounds responsible of the anti-anaphylactic effects of *Impatiens balsamina* L. extracts observed under in vivo assays [53,54,55,56,57,58,59,60].

It is noteworthy that the identification of plant parts with a higher concentration for the mentioned compounds facilitates the selection of plant material for further biological assessments. In addition, based on provided backgrounds, the determination of the selected compounds can be useful to define biological and toxicological biomarkers for the development of products using *I. hawkery* and their quality control.

### 3.2. Determination of TPC, TFC, and TMA Contents

Variations in the polyphenolic constitution of different plant parts have been reported in previous studies for species of *Impatiens* genus and other plants used in healthcare supplements industry (e.g., *Chenopodium quinoa* Willd. and *Calendula officinalis* L.) [37,61,62,63]. This information is also useful in the selection of plant parts to produce extracts with optimized quantities of active compounds for industrial development purposes.

Regarding the effect of drying technique, FL-21 has the best profile for TPC, TFC, and TMA contents followed by OL-21. These results may be explained by the protection of phenolic compounds against thermal degradation provided by drying under low temperatures and the inactivation of polyphenols degradative enzymes exerted by freeze-drying and oven-drying [8,10]. The obtained information shows similar trends to data previously reported for other medicinal plants (e.g., *Backhousia citriodora* F.Muell, *Urtica dioica* L., and *Camellia sinensis* (L.) Kuntze) [64,65,66].

### 3.3. Determination of In Vitro Antioxidant Activity Using Chemical Assays

Polyphenols can exert antioxidant activity through different chemical mechanisms: hydrogen atom transfer (HAT), single electron transfer (SET), and chelating activity over transition metal ions implicated in Fenton and Haber–Weiss reactions involved in the generation of reactive oxygen species (ROS) in biological systems [67,68,69]. ORAC, FRAP, and ferrous iron chelating activity (FICA) assays evaluate the antioxidant activity due to HAT, SET, and chelating activity, respectively, whereas DPPH radical scavenging activity assay is useful to evaluate activity due to combination of SET and HAT mechanisms [67,70]. Table 5 shows the results from the evaluation of the antioxidant activity of extracts from different plant parts of *I. hawkeri* using the mentioned assays. As other plants of *Impatiens* genus, there are variations in the antioxidant activity of the extracts obtained from different parts, a feature that is shared with other medicinal plants and crops (e.g., *Calendula officinalis* and *Chenopodium quinoa*) [26,61,62,63].

Interestingly, data from antioxidant assays applied to the leaf extracts from material dried by different techniques have the same trends observed for TPC and TFC contents (Table 4), except for FICA assay. This evidence supports the role of polyphenols as major antioxidant compounds in *Impatiens* genus plants. Differences in the qualitative and quantitative composition of polyphenols in the extracts might explain the observed differences between the results of chemical antioxidant assays. Individual components in the extracts from different plant parts or from leaves dried in different conditions can act at distinct levels by each chemical antioxidant mechanism.

Chemical transformation of plant components during oven-drying and shade-drying can explain the lowest chelating activity observed in FL-21. We propose that thermal energy or action of enzymes in plant material can modify the hydroxylation pattern of polyphenols enhancing the chelating activity of the extracts. This is based on the fact that polyphenols’ hydroxylation pattern defines their chelating activity [69]. Furthermore, the variability observed in the chromatograms (Figure 1) of the leaf extracts demonstrates that the drying technique influences their chemical constitution. It is noteworthy that data from Table 4 show an increase in the content of kaempferol and quercetin aglycones with shade-drying and oven-drying in comparison to freeze-drying, a finding that may be explained by an increase in flavanol glycoside hydrolysis during drying. This change can promote the exposition of hydroxyl groups that can participate in the formation of complexes with iron (II) ions.

Regarding the trends observed in the results of the chemical antioxidant activity assays for the extracts derived from *I. hawkeri* leaves collected in February 2021, they show a similar pattern than data previously reported for plants of interest for food and pharmaceutical industry such as *Backhousia citriodora* and *Myrtus communis* L. For the first plant, freeze-drying promotes higher antioxidant activity than shade-drying and hot-air-drying. For the second one, oven-drying at 70 °C is better than shade-drying in preserving the antioxidant activity of the extracts [66,71].

### 3.4. Determination of In Vitro Antioxidant Activity Using Biological Assays

Lipid peroxidation is a typical hallmark of oxidative stress in biological systems [72]; this process can generate compounds such as malondialdehyde and acrolein that are recognized carcinogens due to their DNA alkylating activity [73,74]. *I. hawkeri* extracts demonstrate that they have the capability to reduce the peroxidation of lipids from mammal cell membranes according to ERYCA and LPO assays. The results share similar trends to data obtained with chemical antioxidant assays, except for FICA assay, suggesting that HAT and SET mechanisms are relevant for the antioxidant activity observed under LPO and ERYCA assays. Furthermore, results from LPO and ERYCA assays indicate that extracts with the highest levels of TPC and TFC tend to be the most active, evidencing the role of polyphenols and flavonoids as antioxidants with biological relevance [75]. The data of both assays demonstrate that freeze-drying is the best technique to preserve polyphenols and antioxidant activity.

Before the performance of IROS assay, we evaluated the toxicity of the extracts using the resazurin reduction assay (for details, see Figure S6 in Supplementary Material File). The results from this preliminary assay demonstrate that half-maximal inhibitory concentration (IC_50_) on cell viability is higher than 100 µg/mL. None of the extracts reduce the cell viability of Vero cells used in the IROS assay more than 20% when tested at 100 µg/mL. Thus, none of the extracts showed any relevant cytotoxicity [76]. The IROS assay can measure the intracellular antioxidant activity after the exposition of cells to antioxidants. According to the results from this assay, WPE-20 has the highest antioxidant activity. Differences in data trends observed between the IROS assay results and information determined using LPO and ERYCA assays can be explained by the fact that the IROS assay is influenced by additional factors such as the capability of antioxidant compounds to permeate the cell membrane and their modulatory activity on cellular antioxidant signaling pathways [77,78]. Other factors such as synergism and antagonism among plant extract components can contribute to observed differences [79]. However, the most important observation derived from the IROS assay results is the demonstration that *I. hawkeri* extracts can provide cellular antioxidant protection. This information along with data provided by the LPO and ERYCA assay indicates that extracts can protect cells not only at extracellular level but also in the intracellular space.

### 3.5. In Vitro Antibacterial Microdilution Assay

Differences among extracts of different parts of the same plant have been documented in other species of *Impatiens* genus as well as in other medicinal plants, including *Hypericum perforatum*, *Diploknema butyracea* (Roxb.) H.J.Lam and others [26,46,80,81]. Distinction in the composition profile among plant parts may be responsible for this observation. This is supported by experimental results. They demonstrate that extracts from flowers and leaves, which have the highest TFC, TPC, and rutin contents, are the only ones with antibacterial activity.

The results from Table 7 and Table 8 show that extracts with antibacterial effect have an activity that is restricted to Gram-positive bacteria. This trend is consistent with results previously reported for the extracts from other *Impatiens* species [25,26,39,82]. Previous studies have reported that it is more probable to find plant extracts active against Gram-positive than Gram-negative bacteria [83]. The existence of an outer lipopolysaccharide membrane in Gram-negative bacteria constitutes an additional permeability barrier that can hinder the access of bioactive components of the extracts into microorganisms limiting their antibacterial effects [84,85].

In the case of phenolic-rich extracts, polyphenols can exert antibacterial effects due to the disruption of cell membrane permeability; for this mechanism of action, it is necessary to ensure appropriate interactions between hydrophobic motifs of polyphenols and lipophilic components of bacterial cell membrane. However, it is proposed that the outer lipopolysaccharide membrane in Gram-negative bacteria can reduce the cell surface hydrophobicity, giving hinderance to appropriate hydrophobic interactions between polyphenols and cell membrane components. As a consequence, there is a decline in the concentration of polyphenols on the proximity of cell membranes and a reduction in their antibacterial effects in Gram-negative bacteria in comparison to Gram-positive bacteria [86].

HPTLC assays support the presence of phenolic compound groups recognized by their antimicrobial activities among *I. hawker* leaf and flower extracts such as flavonoids (i.e., kaempferol, quercetin, isoquercetin, and rutin), coumarins (i.e., scopoletin), naphtoquinoes (i.e., lawsone), and anthocyanins. Based on the composition profile of studied extracts, it is possible to suggest potential mechanisms responsible for their antibacterial activity. In general, polyphenols with metal chelating activity can interact with metal ions, reducing the availability of essential minerals for cell bacteria metabolism [87]. Chelating antibacterials can also interact with relevant metabolic metalloenzymes, reducing their activity [87,88]. In the specific case of flavonoids, their antibacterial activity can be explained by their inclusion on cell membrane and the disruption of their permeability. Consequently, functions of cell membrane such as osmoregulation, cell respiration, transport processes, the biosynthesis and cross-linking of peptidoglycan, and lipid biosynthesis are compromised [87,89]. Coumarins can bind DNA gyrase in bacteria, resulting in the inhibition of DNA supercoiling [90]. It is proposed that naphthoquinones exert antibacterial activities by three relevant mechanisms: ROS formation due to their conversion to hydroquinone in a nicotinamide adenine dinucleotide (NADH) and molecular oxygen dependent process, metal chelation, and the disruption of cell membrane permeability [91]. Like other flavonoids, anthocyanins can also damage the bacterial cell membrane; in addition, this kind of compound can promote the disruption of the cell wall integrity [92]. As *I. hawkeri* flower and leaf extracts are complex mixtures of flavonoids, coumarins, naphthoquinones (present in FL-21 and SL-21), and anthocyanins (present in LE-20, FE-20, and FL-21), it is expected that they exert antibacterial effects due to a combination of mentioned mechanisms.

It is noteworthy that susceptible Gram-positive bacteria, corresponding to *S. aureus*, *S. epidermidis*, and *E. faecalis*, are recognized foodborne pathogens present in vegetables, dairy products, and meat [93,94,95,96,97]. These results and data from antioxidant activity assessment support the potential use of *I. hawkeri* as a source of natural preservatives as the case of other the extracts from other *Impatiens* species [28,29,30].

### 3.6. Correlation and Principal Component Analysis

Significant correlations of quercetin and rutin contents with results from antioxidant assays are consistent with previous publications that demonstrate the relevant antioxidant activity of these metabolites according to chemical and biological assessments [17,98,99]. The same is true for the significant correlation between rutin content and AAI considering that rutin is a known antibacterial flavonoid [100,101].

Furthermore, the significant correlations of TPC, TFC, and SPC with most of the chemical and biological antioxidant activity assays and AAI index suggest that phenolics are a highly relevant contributor to the evaluated activities. Interestingly, the results from ORAC, DPPH, and FRAP assays demonstrated significant correlations with data from ERYCA and LPO. This finding suggests that the radical scavenging activity of *I. hawkeri* extracts through SET and HAT mechanisms is a key factor that contributes to their antioxidant activity observed under the mentioned biological antioxidant activity assays.

The score plot from PCA (Figure 4b) shows that the extracts can be classified into two main categories corresponding to (1) extracts with high antioxidant activity with antibacterial effect, and (2) extracts with low antioxidant activity without antibacterial effect. The extracts of the first category are in the positive-values zone of PC1, whereas those of the second category are in the negative-values area of the same component. The extracts of the first category are derived from the flowers and leaves of *I. hawkeri,* while those of the second category are extracts from the remaining parts of the plant.

It is remarkable that observed variations on evaluated bioactivities according to the plant part used show consistency with previous reported data for other *Impatiens* plants. Kang et al. have reported that ethanol extracts from *I. balsamina* leaves have higher TPC and TFC values, better antibacterial activities, and more potent DPPH radical scavenging activity in comparison to stem extracts [26]. Chua has evaluated the antioxidant activity of *I. balsamina* stem and leaf methanol extracts. The results from this study demonstrate that leaf extract has higher TPC and TFC values and better performance on DPPH, FRAP, and FICA assays than other analyzed extracts [37].

Nevertheless, there are also some contrasts with previously published data for other *Impatiens* species. The comparison of *I. balsamina* flower, leaf, and stem ethanol extracts indicates that leaf and flower extracts exert higher activity than stem extract with higher levels for TPC and TFC; however, flower extract has higher TPC and TFC levels and better performance on DPPH radical scavenging assay than leaf extract [102]. Methanol extracts from *Impatiens glandulifera* Royle roots tend to be more potent than flower and leaf extracts from the same plant according to DPPH and FICA assays [38]. The comparative analysis of *Impatiens chinensis* L. seed, leaf, stem, root, and flower methanol extracts demonstrated that flower extract has the highest DPPH radical scavenging activity and TPC value followed by seed, leaf, stem, and root extracts [63].

In the case of medicinal plants from other genera, variability in the antioxidant activity of the extracts according to used plant part has been also reported. For instance, the comparative evaluation of the antioxidant activity of *Calendula officinalis* methanol extracts prepared by maceration shows that flower extract has higher DPPH radical scavenging activity and FRAP, TPC, and TFC values in comparison to leaf and root extracts; however, leaf extract demonstrates better results under FICA assay [61]. Another example is *Chenopodium quinoa*, the flower ethanol extracts prepared with air-dried plant material harvested in the third month after seeding have higher TPC and TFC values than leaf, root, and stem extracts; nonetheless, root extracts tend to have better antioxidant activity according to DPPH and FRAP assays [62]. Evidence regarding the identification of variations in the antibacterial activity of extracts according to the plant part used for their preparation has been published for medicinal plants including *Hypericum perforatum*; the root methanol extract from this plant tends to lower MICs in comparison to plant shoots [46]. *Diploknema butyraceais* is another example of antibacterial activity variation related to plan part selection; the methanol root bark extract and the ethyl acetate pericarp extract have shown a wider antibacterial spectrum and lower MICs in comparison to leaf extracts [80]. These backgrounds support the necessity of studies dealing with plant part influence on the bioactivity profile of medicinal plant material in order to get clues for the optimization of the biological effects of potential healthcare products or natural preservatives.

Moreover, significant correlations between the content of phenolic compounds and the antioxidant and antibacterial activities of extracts have been previously reported for other medicinal plants (e.g., *Rhus verniciflua* Stokes and *Papaver rhoeas* L.) [103,104]. In the case of extracts from plants of *Impatiens* genus, the existence of a significant correlation between their contents of phenolic compounds and antioxidant activity has previously been reported; however, no significant correlation has been reported with their antibacterial activity according to the reviewed literature [25,26].

### 3.7. Further Directions Regarding Practical Applications

As discussed above, obtained data give support for further research on the evaluation of additional biological activities of *I. hawkeri* extracts taking into account the bioactivity profile of targeted compounds and their differential distribution on plant parts. Considering the implication of oxidative stress in inflammation, cancer development, cardiovascular diseases, and neurodegenerative pathologies, we propose further studies dealing with the evaluation of *I. hawkeri* extracts as a potential agent for the prevention of mentioned health problems using proper in vitro and in vivo experiments in order to obtain insights related to the extract mechanism of actions. In this way, it will be possible to develop healthcare products using *I. hawkeri* taking advantage of the cumulative experience in the propagation and cultivation of this plant that has been currently used just as an ornamental plant at the industrial level. The determination of targeted compounds and the correlation analysis of their concentration with biological effects provide information for the selection of chemical biomarkers for the quality control of potential products developed with *I. hawkeri* as starting material.

In the short term, it will be worth analyzing the potential of *I. hawkeri* extracts as food preservatives. Medicinal plant extracts rich in polyphenolics have been used as food preservatives for products such as meat, dairy products, bread, among others, taking advantage of their antimicrobial and antioxidant activities and best toxicological profile in comparison to common chemical preservatives [4,86]. For instance, carboxymethyl cellulose coatings formulated with *Impatiens balsamina* stem extracts have demonstrated to be effective in decreasing the decay rate and weight loss of citrus fruits. These coatings also provide protection to spoilage microorganisms [28,29]. However, it is important to highlight the necessity of a complete toxicological characterization of *I. hawkeri* extracts to gain a clear understanding of their applicability, even though this species has been used as a medicinal plant [34,35,36].

## 4. Materials and Methods

### 4.1. Chemicals and Reagents

HPTLC-grade 60 F254 or 60 F254s silica gel plates (20 × 10 cm), quercetin, kaempferol, rutin, isoquercetin, lawsone, 2-methoxy-1,4-naphthoquinone (2-MNQ), scopoletin, gallic acid, 6-hydroxy-2,5,7,8-tetramethylchroman-2-carboxylic acid (known as trolox), fluorescein disodium salt, 2′,7′-dichlorodihydrofluorescein diacetate (H2DCFDA), 2,2-diphenyl-1-picrylhydrazyl (DPPH), 2,2′-azobis(2-amidinopropane) dihydrochloride (AAPH), *tert*-Butyl hydroperoxide (TBHP), thiobarbituric acid (TBA), 3-(2-pyridyl)-5,6-diphenyl-1,2,4-triazine-*p*,*p*′-disulfonic acid monosodium salt hydrate (known as ferrozine), iron (III) chloride hexahydrate, iron (II) chloride tetrahydrate, Folin–Ciocalteu 2 N reagent, sodium nitrite, formic acid (88% *w*/*w*), 2-butanol, penicillin/streptomycin (10,000 U/10 mg per mL), resazurin sodium salt, L-glutamine, amphotericin B, trypsin, fetal bovine serum, Dulbecco’s Modified Eagle medium (DMEM) without phenol red, cation adjusted Müller–Hinton broth, tryptic soy agar, and trizma base were purchased from Sigma-Aldrich (Saint Louis, MO, USA). Toluene, *n*-hexane, acetic acid, chloroform, methanol, hydrochloric acid, sodium carbonate, and aluminum chloride hexahydrate were obtained from Merck (Darmstadt, Germany). Ethyl acetate, potassium ferricyanide, ethylenediaminetetraacetic acid (EDTA) disodium salt dihydrate, and trichloroacetic acid were supplied by J.T. Baker (Phillipsburg, NJ, USA). Other reagents were obtained from different suppliers: ethanol (FANAL, Grecia, Costa Rica), *n*-butanol (Fischer Chemical, Fair Lawn, NJ, USA), minimum essential medium Eagle (Gibco, Grand Island, NY, USA), brain heart Infusion agar (BD Difco, Sparks, MD, USA), brain heart infusion broth (BD Difco, Sparks, MD, USA), ciprofloxacin chloride (USP, Rockville, MD, USA), and ceftriaxone disodium salt (USP, Rockville, MD, USA).

### 4.2. Plant Material Collection

Plant material has been identified as previously reported with the deposit of a voucher specimen at Dr. Fournier Origgi herbarium, School of Biology, Universidad de Costa Rica. The assigned collection code corresponds to USJ 110 989 [39]. *I. hawkeri* material was purchased at a local plant nursery in Grecia, Alajuela province, Costa Rica. Two collections were carried out on different dates. The first collection was in February 2020. The material was divided into six groups corresponding to whole plants, aerial parts (mixture of leaves, stems, and flowers), leaves, flowers, stems, and roots. The materials from the first collection were oven-dried at 70 ± 4 °C (Heratherm, natural convection, Thermo Scientific, Waltham, MA, USA) except for flowers that are delicate for thermal treatment; in this case, they were freeze-dried at −50 ± 2 °C and 0.100 mBar (Labconco, Freezone 6, Kansas City, MO, USA). The second collection was carried out in February 2021, due to the availability of plant material and the relative high antioxidant and antibacterial activities of the leaf extract in comparison to the extracts from other plant parts of *I. hawkeri* (for details, see Results and Discussion sections), the material from this collection was leaves exclusively. It was divided into three equal portions, and each one was dried by different drying techniques: freeze-drying, oven-dying at 70 ± 4 °C, and shade-drying at room temperature (24 ± 7 °C). After drying, the plant material of both collections was grinded with a commercial food processor and sieved to the highest particle size of 2 mm. Data of weight loss on drying are reported in Table S2 of the Supplementary Material.

### 4.3. Extract Preparation

Plant extracts of each plant material were prepared by maceration according to the procedure described by Kang et al. [26] with modifications. The ethanol–water mixture used as extraction solvent had a concentration of 80% *v*/*v*. The plant material–solvent ratio was 1:10 and 1:15 for the first and the second maceration steps, respectively. The first maceration step lasted two weeks, and the time for the second maceration period was a week. The maceration process was carried out under light protection. After preparation, the extracts were freeze-dried and stored at −80 °C until their analysis. Six extracts were obtained from material collected in February 2020: whole-plant extract (WPE-20), aerial-part extract (APE-20), leaf extract (LE-20), flower extract (FE-20), stem extract (SE-20), and root extract (RE-20), whereas three extracts were produced from leaves obtained during February 2021: freeze-dried leaf extract (FL-21), oven-dried leaf extract (OL-21), and shade-dried leaf extract (SL-21). Data of extraction yield are reported in Table S3 of Supplementary Material.

### 4.4. Quantitative Estimation of Bioactive Polyphenols by High-Performance Thin-Layer Chromatography (HPTLC)

#### 4.4.1. General Chromatographic Conditions

Standard solutions (SSs) of each analyzed compound were accurately prepared to adequate concentration using methanol as solvent. Extract solutions were prepared in the same solvent at concentration of 10 mg/mL. The standards and extracts solutions were transferred to autosampler amber vials; then, they were applied in spray mode, using nitrogen as propellent gas (applying velocity 150 nL/s) in the form of bands on HPTLC grade silica gel plates using the ATS4 autosampler (CAMAG, Muttenz, Switzerland). Bands had 8 mm length and 1 mm thickness. Each band was separated from others by 3.4 mm. Distance between the lateral left border of the plate and the first band was fixed in 20 mm. The solvent front was adjusted to 80 mm from the plate bottom. After applying standards and sample solutions to the plate, it was subjected to pre-drying for 30 s using the automatic developing chamber ADC2 (CAMAG, Muttenz, Switzerland). Then, the developing chamber was saturated with mobile phase. After saturation time, the plate was developed and dried for 5 min. The chromatographic development of plates was carried out at room temperature (24 ± 3 °C) and relative humidity (51 ± 3%). Images from the chromatograms were obtained under visible or UV light (254 nm or 366 nm wavelength); for this step, TLC Visualizer (CAMAG, Muttenz, Switzerland) was used. Densitograms of developed plates were measured using TLC Scanner 4 (CAMAG, Muttenz, Switzerland). For determinations in absorbance mode, deuterium lamp was used, whereas fluorescence determinations were established using mercury lamp as excitation light source and K400 filter to measure the emitted fluorescence. Scanning velocity, resolution, and slit size were set at 20 mm/s, 100 µm/step, and 4 × 0.1 mm, respectively. Chromatograms and densitograms were processed and analyzed using VisionCATS 3.1 software (CAMAG, Muttenz, Switzerland). Absorbance and fluorescence data were measured at reference wavelengths and retention factor (Rf) ranges corresponding to each standard. Peak height data from standard bands were used to build calibration curves by linear regression; from there, concentration of each compound in the extracts was calculated. Each determination was measured in triplicate, and results are reported as average concentration (mg/g of extract dry weight (DW)) with the corresponding standard deviation. Additionally, the sum of polyphenol compound concentration (SPC) determined by HPTLC analyses was reported for each extract. Analytical parameters of each chromatographic method are reported in Table S4 of Supplementary Material. Sample densitograms are available in the same additional document (Figures S1–S5).

#### 4.4.2. Simultaneous Determination of Quercetin and Kaempferol

Linearity ranges were 120–480 ng/band and 100–400 ng/band for quercetin and kaempferol, respectively. For the determination of both compounds in FE-20, mobile phase 1 (toluene–ethyl acetate–formic acid (60:45:3 *v*/*v*/*v*)) was used with a saturation time of 10 min. For the other extracts, a double development system was used, chromatographic plate was first developed with mobile phase 1 after 10 min of saturation, and then the plate was dried and consecutively developed with mobile phase 2 (toluene–ethyl acetate–*n*-hexane–formic acid (60:30:10:3 *v*/*v*/*v*/*v*)) after 10 min of saturation. Different conditions applied to FE-20 are due to the better separation of component bands observed to it with the single-step development system in comparison to the double-step development system. After chromatographic development steps, the plate was dried and visualized under UV light (366 nm wavelength). Absorbance densitograms were measured at wavelength of 380 nm. For the analysis of FE-20, the Rf ranges for quercetin and kaempferol were 0.410 ± 0.017 and 0.553 ± 0.010, respectively. For the other extracts, the Rf ranges were 0.504 ± 0.020 and 0.713 ± 0.017, respectively.

#### 4.4.3. Simultaneous Determination of Quercetin 3-O-β-D-Rutinoside (Rutin) and Quercetin 3-O-β-D-Glucopyranoside (Isoquercetin)

Linearity range for rutin and isoquercetin was 280–980 ng/band. For the analysis of the extracts, the chromatographic plate was developed with mobile phase 3 (butan-2-ol–*n*-butanol–ethyl acetate–formic acid (60:40:15:10 *v*/*v*/*v*/*v*)) after 20 min of saturation. After development, the plate was dried and visualized under UV light (254 nm wavelength). Absorbance densitograms were measured at wavelength of 365 nm. The Rf ranges for rutin and isoquercetin were 0.346 ± 0.023 and 0.620 ± 0.021, respectively.

#### 4.4.4. Simultaneous Determination of 2-Hydroxy-1,4-Naphthoquinone (Lawsone) and 2-Methoxy-1,4-Naphtoquinone (2-MNQ)

Linearity ranges were 220–440 ng/band and 340–640 ng/band for lawsone and 2-MNQ, respectively. A double development system was used, chromatographic plate was first developed with mobile phase 4 (toluene–ethyl acetate–acetic acid (80:30:3 *v*/*v*/*v*)) after 10 min of saturation, and then the plate was dried and consecutively developed with mobile phase 5 (toluene–ethyl acetate–*n*-hexane–acetic acid (80:30:20:10 *v*/*v*/*v*/*v*)) after 10 min of saturation. After chromatographic development steps, the plate was dried and visualized under UV light (254 nm wavelength). Absorbance densitograms were measured at wavelength of 275 nm. The Rf ranges for lawsone and 2-MNQ were 0.719 ± 0.018 and 0.848 ± 0.010, respectively.

#### 4.4.5. Determination of 7-Hydroxy-6-Methoxycoumarin (Scopoletin)

Linearity range was 30–300 ng/band. A double development system was used, chromatographic plate was first developed with mobile phase 6 (chloroform–ethyl acetate–formic acid (60:30:10 *v*/*v*/*v*)) after 10 min of saturation, and then the plate was dried and consecutively developed with mobile phase 7 (chloroform–ethyl acetate (60:40 *v*/*v*)) after 10 min of saturation. After chromatographic development steps, the plate was dried and visualized under UV light (366 nm wavelength). Fluorescence densitograms were obtained using an excitation wavelength of 302 nm, and the emitted fluorescence was measured with K400 filter. The Rf range for scopoletin was 0.623 ± 0.037.

### 4.5. Determination of Total Phenolic Content (TPC)

TPC values of the extracts were determined according to 96-well microplate Folin–Ciocalteu method described by Bobo-Garcia et al. [105]. The absorbance obtained with the standard solutions of gallic acid was corrected by subtracting the absorbance of the blank corresponding to 20 µL of distilled water subjected to the analysis procedure. In the case of the extracts, the obtained absorbance was corrected by subtracting the absorbance of their corresponding sample blank: 20 µL of extract test solution treated with the analysis procedure adding water instead Folin–Ciocalteu reagent. This procedure allows one to avoid the interference produced by any intrinsic absorbance from extract solutions. Each determination was measured in triplicate, and results are reported as average concentration of gallic acid equivalents (GAEs) (mg GAE/g of extract DW) with the corresponding standard deviation.

### 4.6. Determination of Total Flavonoid Content (TFC)

Total flavonoid contents (TFCs) were determined using the aluminum chloride method described by Magalhães et al. [106] with modifications. Quercetin was used as reference flavonoid. The standard solutions were prepared in methanol with a concentration range of 20–60 µg/mL. The absorbance data from quercetin standard solutions were corrected by subtracting the absorbance of their blank corresponding to 50 µL of methanol subjected to the analysis procedure. In the case of the extracts, to avoid interferences due to their intrinsic color, absorbance values were corrected by subtracting the absorbance of their corresponding sample blank: 50 µL of extract test solution treated with the analysis procedure adding water instead aluminum chloride reagent. Each determination was measured in triplicate, and results are reported as average concentration of quercetin equivalents (QEs) (mg QE/g of extract DW) with the corresponding standard deviation.

### 4.7. Determination of Total Monomeric Anthocyanins (TMA)

TMA was determined according to pH differential method described by Leeet al. [107]. Each determination was measured in triplicate, and results are reported as average concentration of cyanidin 3-*O*-glucoside equivalents (C3GE) (mg C3GE/g of extract DW) with the corresponding standard deviation.

### 4.8. In Vitro Chemical Antioxidant Assays

#### 4.8.1. Oxygen Radical Absorbance Capacity Assay (ORAC)

ORAC values of the extracts were determined according to the procedure described by Kenny, Brunton, and Smyth [108] with minor modifications. Under this method, antioxidant compounds from the extracts reduce the fluorescence decay rate of fluorescein exposed to free radicals generated from 2,2′-azobis(2-amidinopropane) dihydrochloride (AAPH). Fluorescence decay curves for standard and sample solutions wells were recorded for 60 min. Trolox calibration curve was constructed with a linearity range of 20–60 µM. Quercetin was used as positive control. Each determination was measured in triplicate, and the antioxidant activity is reported as average concentration of trolox equivalents (TE) (mmol TE/g of extract DW) with the corresponding standard deviation.

#### 4.8.2. DPPH Radical Scavenging Activity Assay

The concentration of the extract, quercetin (positive control), or trolox (reference antioxidant) that scavenges the half of DPPH radical concentration (IC_50DPPH_) was determined according to the 96-well microplate method described by Kenny, Brunton, and Smyth [108] with minor modifications. The absorbance values from quercetin test solutions were corrected by subtracting the absorbance of their blank corresponding to 100 µL of methanol. In the case of the extracts, to avoid interferences due to their intrinsic color, absorbance data were corrected by subtracting the absorbance of their corresponding sample blank: a mixture of 50 µL of extract test solution and 50 µL of methanol instead of DPPH reagent. The IC_50DPPH_ for each substance was calculated by interpolation in the Hill equation of the corresponding dose-response curve [109]. The trolox equivalent antioxidant capacity (TEAC) was calculated using IC_50DPPH_ data according to Shimamura et al. [110]. Each determination was measured in triplicate, and the antioxidant activity is reported as average concentration of trolox equivalents (TE) (mmol TE/g of extract DW) with the corresponding standard deviation.

#### 4.8.3. Ferric Reducing Antioxidant Power Assay (FRAP)

FRAP values of the extracts were determined adapting the procedure described by Işıl Berker et al. [111] to 96-well microplate method. The method is based on the reduction of ferricyanide to ferrocyanide giving a colored complex in the presence of free iron (III) ions whose absorbance can be monitored at 700 nm. Trolox standard solutions were prepared in methanol with a linearity range of 20–100 µM. Extracts and quercetin (positive control) were adequately diluted to a concentration that allowed the determination of their antioxidant activity by interpolation in the trolox calibration curve. Aliquots of 20 µL of trolox, quercetin, or extracts solutions were dispensed in the wells of the microplate. Then, the following reagents were added in the mentioned order: 130 µL of 30 mM HCl, 25 µL of 1.2% *w*/*v* potassium ferricyanide, and 25 µL of 0.1% *w*/*v* iron (III) chloride hexahydrate. As blank for standard solutions and positive control, 20 µL of methanol was used instead of trolox or quercetin solutions, and the procedure was applied as mentioned before. For extracts, the blank was 20 µL of the corresponding solutions treated with the same reagents, substituting the 25 µL aliquot of 1.2% *w*/*v* potassium ferricyanide by 25 µL of distilled water. In this way, it was possible to correct the absorbance data considering the intrinsic optical density of the samples. Then, the plate was incubated for 40 min at room temperature inside the microplate reader (Synergy HT, BioTek Instruments, Winooski, VT, USA), and the absorbance was read at wavelength of 700 nm. Each determination was measured in triplicate, and the antioxidant activity is reported as average concentration of trolox equivalents (TEs) (mmol TE/g of extract DW) with the corresponding standard deviation.

#### 4.8.4. Ferrous Iron Chelating Activity Assay (FICA)

The iron (II) chelating activity of the extracts and quercetin (positive control) was determined according to the method described by Sousa Santos, Alvarenga Brizola, and Granato [67] with minor modifications. The method is based on the formation of complexes between plant extract compounds and iron (II) ions decreasing their availability to interact with ferrozine, an iron (II) chelating compound, promoting a reduction in the absorbance of solutions at wavelength of 562 nm. A solution of iron (II) chloride 0.3 mM was used as the source of iron (II) ions. The EDTA linearity range was 25–100 µM. Each determination was measured in triplicate, and the chelating activity is reported as average concentration of ethylenediaminetetraacetic acid equivalents (EDTAEs) (µmol EDTAE/g of extract DW)) with the corresponding standard deviation.

### 4.9. In Vitro Biological Antioxidant Assays

#### 4.9.1. Rat Liver Homogenate Lipid Peroxidation Inhibition Assay (LPO)

The approval for the use of laboratory animals was provided by Institutional Committee for Care and Use of Animals, Universidad de Costa Rica (CICUA), according to permission number CICUA-018-2022. Livers were obtained from 7-week-old male Sprague-Dawley rats (mean weight ± standard deviation: 221 ± 25 g). Before the liver extraction procedure, the animals had one week of acclimatization at 20–30 °C, 60–70% relative humidity, and 12 h light–dark cycles with ad libitum access to food and water. After this period, the animals were anesthetized under carbon dioxide (CO_2_) chamber and euthanized by decapitation followed by removal of liver. LPO assay was carried out on liver homogenates according to the procedures described by Azofeifa et al. [112]. The test concentration ranges were 7.8–2000 µg/mL and 2.8–45 µg/mL for extracts and quercetin (positive control), respectively. The half-maximal inhibitory concentration of the extracts or quercetin (positive control) against rat liver homogenates lipid peroxidation (IC_50LPO_) was determined in quadruplicate (on four different rat liver homogenates). The antioxidant activity is reported as average IC_50LPO_ (µg/mL) with the corresponding standard deviation.

#### 4.9.2. Erythrocyte Cellular Antioxidant Activity Assay (ERYCA)

The capability of the extracts to prevent erythrocyte hemolysis induced by membrane lipid peroxidation was determined by the method described by González et al. [113]. The extracts test concentration ranges were 7.5–60 µg/mL. Each determination was measured in triplicate, and the antioxidant activity is reported as average concentration of quercetin equivalents (QEs) (mmol QE/g of extract DW) with the corresponding standard deviation.

#### 4.9.3. Intracellular Reactive Oxygen Species Inhibition Assay (IROS)

The cytotoxicity of the extracts was evaluated before the determination of IROS capability on African green monkey kidney epithelial cells (Vero cells (ATCC CCL-81)) by resazurin reduction assay according to the method described by Gómez-García et al. with modifications [114]. None of the extract induced a cell viability lower than 80% at highest test concentration of 100 µg/mL as a prerequisite for IROS assay. Details about the method and results of this preliminary cytotoxicity assay are available in the Supplementary Material (Figure S6). The protective activity of the extracts and quercetin (positive control) against intracellular oxygen reactive species generation was determined on the same cell line. Minimum essential medium (MEM) supplemented with phenol red, bovine fetal serum (10% *v*/*v*), L-glutamine (2 mM), penicillin/streptomycin (50 U/mL), and amphotericin B (250 µg/mL) was used as culture media. Cells were prepared in a suspension of 250,000 cells/mL. From this suspension, 100 µL aliquots were aseptically transferred to the wells of a flat-bottom sterile black microplates and incubated at 37 °C for 22 h under 5% CO_2_ atmosphere to promote cell adherence. After this period, the cells were washed with sterile phosphate buffered saline (PBS), and 100 µL of extract (100 µg/mL) or quercetin (24 µg/mL) solutions prepared in culture media was added to each well. In each plate, control wells were reserved for basal fluorescence (cells without reactive oxygen species (ROS) induction) and maximal fluorescence (cells with ROS induction without antioxidant treatment). For these wells, 100 µL of culture media was added instead of extract or quercetin solutions. Subsequently, the plate was incubated at 37 °C for 22 h under 5% CO_2_ atmosphere. After incubation, cells were washed with PBS, and 100 µL of 100 µM 2′,7′-dichlorofluorescein diacetate (H2DCFDA) solution in phenol red free culture medium was added to each well followed by an incubation period of 1 h at 37 °C under 5% CO_2_ atmosphere. Then, wells were washed with PBS, and 100 µL of 150 µM *tert*-butyl hydroperoxide (TBHP) solution in phenol red free culture medium was added to each well except for basal fluorescent control wells; to them, 100 µL of culture media without TBHP was added. The plate was incubated for 15 min, and fluorescence was measured with a microplate reader (Cytation 3, BioTek Instruments, Winooski, VT, USA) at excitation and emission wavelengths of 485 and 535 nm. The IROS percentage *(*% *IROS*) was calculated using the following equation (Equation (1)):

(1) $$\%\;IROS=\frac{\left(M-B\right)-(A-B)}{M-B}\times 100\%$$

where *B* is the average fluorescence of basal fluorescence control, *M* is the average fluorescence of maximal fluorescence control (maximal intracellular ROS production control), and *A* is the average fluorescence of wells treated with extract or quercetin. The antioxidant activity was measured in quintuplicate and reported as average % IROS with the corresponding standard deviation.

### 4.10. In Vitro Antibacterial Microdilution Assay

*Staphylococcus aureus* (ATCC 6538), *Staphylococcus epidermidis* (ATCC 12228), *Enterococcus faecalis* (ATCC 29212), *Pseudomonas aeruginosa* (ATCC 15442), *Escherichia coli* (ATCC BAA-2452), *Klebsiella pneumoniae* (ATCC 10031), and *Salmonella enterica* subsp. *enterica serovar* Typhimurium (known as *S. typhimurium*) (ATCC 14028) were used as test bacteria. For the assays, 22 h cultures in heart infusion agar (*E. faecalis*) or tryptic soy agar (*S. aureus*, *S. epidermidis*, *P. aeruginosa*, *E. coli*, *K. pneumoniae*, and *S. typhimurium*) were used. Inoculums were prepared in sterile culture broth (brain heart infusion broth for *E. faecalis* or cation adjusted Müller–Hinton broth for the remaining strains). These suspensions were used to perform the broth microdilution assays according to the method described by EUCAST [115]. The extracts were tested at a maximum concentration of 10 mg/mL, while positive control (ciprofloxacin chloride for *E. coli* and *E. faecalis* or ceftriaxone disodium salt for other bacteria) was tested at a maximum concentration of 64 µg/mL. The assays were repeated in triplicate over each test bacteria, and the results for the extracts and controls are reported as the average minimum inhibitory concentration (MIC) with the corresponding standard deviation. Additionally, antibacterial activity index (AAI) was calculated for the extracts using the following equation (Equation (2)):

(2) $$AAI=\frac{n}{AMIC}$$

where *n* is the number of susceptible bacteria to extracts, and *AMIC* is the average MIC determined by each extract on all susceptible bacteria. This index was created to have an integrated measure of the overall antibacterial activity in terms of potency and spectrum (number of bacteria susceptible to each extract) for the purpose of the correlation analyses described in the next section. *AAI* is higher when MICs are low, and the number of susceptible test strains increases.

### 4.11. Statistical Analysis

Kinetic data analysis for ORAC and ERYCA assay was carried out using Gen 5 3.09 software (BioTek Instruments, Winooski, VT, USA). Calculations of IC_50_, average results, and standard errors were made with GraphPad Prism 9.4 (San Diego, CA, USA). This software was also used to perform Student’s *t*-tests, one-way analysis of variance (ANOVA) (simple ANOVA for data without different variances or Welch’s ANOVA for data with different variances), and *post-hoc* tests. For multiple comparisons, contrasts between the results of extracts and controls were determined by *Dunnett’s* test. For the comparison of results between extracts, Tukey’s test (for data without different variances) or T3 Dunnett’s test (for data with different variances) was used. Spearman’s rank correlation analysis was made using GraphPad Prism 9.4. Principal component analysis (PCA) with unit standardization was carried out with STATISTICA 8.0 software (StatSoft, Inc., Tulsa, OK, USA). For statistical analyses, a significance level of α = 0.05 was used.

## 5. Conclusions

Data suggest that quercetin, kaempferol, rutin, isoquercetin, scopoletin, and lawsone, bioactive polyphenols previously reported in *Impatiens* plants, are present among the *I. hawkeri* extracts analyzed in the present research work. Furthermore, flower and leaves are the parts of *I. hawkeri* that allow the production of extracts with the highest antioxidant and antibacterial activity according to most of the experimental models. The evidence of antioxidant activity in biological assays is remarkable as few plants of *Impatiens* genus have demonstrated activity through this kind of methods previously. Freeze-drying is the best drying technique to optimize the antioxidant activity of *I. hawkeri* leaf extracts, whereas oven-dying was the technique that allowed the production of *I. hawkeri* leaf extract with the best antibacterial profile. Further studies of extraction and fractionation techniques can be carried out to maximize the antioxidant activity and to enhance the antibacterial activity observed for *I. hawkeri* leaf extracts.

Another relevant conclusion is the fact that the contents of quercetin and rutin and TPC, TFC, ORAC, FRAP, and DPPH parameters are quantitative features of *I. hawkeri* extracts significantly correlated with activities evaluated under in vitro biological models. Considering the low complexity of their determination, we propose that they may be used as indicators to drive further studies for the improvement of *I. hawkeri* extract activities. Additionally, the relevant antioxidant activity demonstrated by leaf, flower, and whole-plant extracts on biological assays can support further research to evaluate their effects on experimental models of oxidative-stress-related pathologies, such as inflammation, cancer development and progression, and neurodegenerative illnesses. Finally, the results obtained demonstrate that *I. hawkeri*, a well-known species in ornamental plant industry, can be considered as a novel source of antioxidants, antibacterials, and known bioactive polyphenols for potential applications in other industries, including the development of polyphenol-rich nutraceuticals and natural preservatives. Considering that the present work is based on in vitro assays, further experiments using in vivo models and food matrices are necessary to confirm these applications.

## Figures and Tables

**Table 1:** Content of bioactive polyphenols in the ethanol extracts from different plant parts of *Impatiens hawkeri*.

Extract	Content (mg/g of Extract Dry Weight)					
	Quercetin	Kaempferol	Rutin	Isoquercetin	Scopoletin	SPC
WPE-20	N.D	0.19 ± 0.02 ^A^	N.D	N.D	0.47 ± 0.05 ^A^	0.66
APE-20	0.42 ± 0.02 ^A^	0.30 ± 0.02 ^B^	2.68 ± 0.12 ^A^	N.D	0.78 ± 0.03 ^B^	4.18
LE-20	0.17 ± 0.02 ^B^	N.D	4.58 ± 0.19 ^B^	1.65 ± 0.19	1.35 ± 0.07 ^C^	7.75
FE-20	0.03 ± 0.02 ^C^*	0.17 ± 0.02 ^A^	23.80 ± 1.74 ^C^	N.D	0.05 ± 0.01 ^D^	24.05
SE-20	N.D	N.D	0.24 ± 0.05 ^D^	N.D	0.41 ± 0.06 ^A^	0.65
RE-20	N.D	N.D	N.D	N.D	2.24 ± 0.07 ^E^	2.24

All values are mean ± standard deviation (SD) of three replicates. Different superscript letters in the same column indicate significant differences (*p* < 0.05). * Estimation is lower than quantification limit. SPC: sum of polyphenols determined by HPTLC; N.D: not detected; WPE-20: whole-plant extract from material collected in 2020; APE-20: aerial-part extract from material collected in 2020; LE-20: leaf extract from material collected in 2020; FE-20: flower extract from material collected in 2020; SE-20: stem extract from material collected in 2020; RE-20: root extract from material collected in 2020.

**Table 2:** Content of bioactive polyphenols in the ethanol extracts from *Impatiens hawkeri* leaves dried by different techniques.

Extract	Content (mg/g of Extract Dry Weight)						
	Quercetin	Kaempferol	Rutin	Isoquercetin	Lawsone	Scopoletin	SPC
FL-21	0.65 ± 0.05 ^A^	0.26 ± 0.02 ^A^	5.51 ± 0.28 ^A^	2.88 ± 0.24	0.99 ± 0.07 ^A^	0.81 ± 0.03 ^A^	11.10
OL-21	2.03 ± 0.17 ^B^	0.76 ± 0.02 ^B^	9.74 ± 0.55 ^B^	N.D	N.D	1.42 ± 0.09 ^B^	13.95
SL-21	2.72 ± 0.14 ^C^	1.70 ± 0.05 ^C^	2.53 ± 0.14 ^C^	N.D	0.13 ± 0.01 ^B^	0.16 ± 0.02 ^C^	7.24

All values are mean ± SD of three replicates. Different superscript letters in the same column indicate significant differences (*p* < 0.05). SPC: sum of polyphenols determined by HPTLC; N.D: not detected; FL-21: freeze-dried leaf extract from material collected in 2021; OL-21: oven-dried leaf extract from material collected in 2021; SL-21: shade-dried leaf extract from material collected in 2021.

**Table 3:** Total phenolic content (TPC), total flavonoid content (TFC), and total monomeric anthocyanins content (TMA) for the ethanol extracts from different plant parts of *Impatiens hawkeri*.

Extract	TPC (mg GAE/g DW)	TFC (mg QE/g DW)	TMA (mg C3GE/g DW)
WPE-20	15.59 ± 0.54 ^A^	23.40 ± 2.68 ^A^	N.D
APE-20	37.79 ± 1.57 ^B^	57.97 ± 3.46 ^B^	N.D
LE-20	156.49 ± 3.10 ^C^	333.66 ± 11.45 ^C^	0.14 ± 0.01 ^A^
FE-20	124.75 ± 6.80 ^D^	100.48 ± 6.95 ^D^	4.96 ± 0.14 ^B^
SE-20	7.50 ± 0.21 ^A^	11.33 ± 0.28 ^A^	N.D
RE-20	14.83 ± 0.69 ^A^	28.88 ± 3.32 ^A,E^	N.D

All values are mean ± SD of three replicates. Different superscript letters in the same column indicate significant differences (*p* < 0.05). DW: extract dry weight; N.D: not detected; GAE: gallic acid equivalents; QE: quercetin equivalents; C3GE: cyanidin 3-*O*-glucoside equivalents; WPE-20: whole-plant extract from material collected in 2020; APE-20: aerial-part extract from material collected in 2020; LE-20: leaf extract from material collected in 2020; FE-20: flower extract from material collected in 2020; SE-20: stem extract from material collected in 2020; RE-20: root extract from material collected in 2020.

**Table 4:** Total phenolic content (TPC), total flavonoid content (TFC), and total monomeric anthocyanins content (TMA) for the ethanol extracts from *Impatiens hawkeri* leaves dried by different techniques.

Extract	TPC (mg GAE/g DW)	TFC (mg QE/g DW)	TMA (mg C3GE/g DW)
FL-21	342.66 ± 4.34 ^A^	979.96 ± 43.34 ^A^	0.29 ± 0.02
OL-21	232.10 ± 5.07 ^B^	534.03 ± 8.53 ^B^	N.D
SL-21	165.93 ± 0.90 ^C^	344.70 ± 11.30 ^C^	N.D

All values are mean ± SD of three replicates. Different superscript letters in the same column indicate significant differences (*p* < 0.05). DW: extract dry weight; N.D: not detected; GAE: gallic acid equivalents; QE: quercetin equivalents; C3GE: cyanidin 3-*O*-glucoside equivalents; FL-21: freeze-dried leaf extract from material collected in 2021; OL-21: oven-dried leaf extract from material collected in 2021; SL-21: shade-dried leaf extract from material collected in 2021.

**Table 5:** In vitro chemical antioxidant activity of the ethanol extracts from different plant parts of *Impatiens hawkeri*.

Extract	ORAC (mmol TE/g DW)	DPPH (mmol TE/g DW)	FRAP (mmol TE/g DW)	FICA (µmol EDTAE/g DW)
WPE-20	0.69 ± 0.07 ^A^	0.05 ± 0.00 ^A^	0.08 ± 0.01 ^A^	43.50 ± 2.51 ^A,B^
APE-20	1.52 ± 0.03 ^B^	0.14 ± 0.00 ^A^	0.20 ± 0.02 ^B^	39.90 ± 1.47 ^A^
LE-20	3.65 ± 0.02 ^C^	0.81 ± 0.06 ^B^	0.74 ± 0.02 ^C^	50.76 ± 3.04 ^B^
FE-20	4.27 ± 0.07 ^D^	0.64 ± 0.06 ^C^	0.62 ± 0.02 ^D^	3.74 ± 0.12 ^C^
SE-20	0.36 ± 0.02 ^E^	0.05 ± 0.00 ^A^	0.06 ± 0.00 ^A^	36.00 ± 4.45 ^A^
RE-20	0.47 ± 0.01 ^A,E^	0.06 ± 0.00 ^A^	0.08 ± 0.00 ^A^	39.12 ± 3.14 ^A^
Control (quercetin)	23.11 ± 0.83 ^F^	5.87 ± 0.75 ^D^	12.53 ± 1.06 ^E^	23.32 ± 0.83 ^D^

All values are mean ± SD of three replicates. Different superscript letters in the same column indicate significant differences (*p* < 0.05). ORAC: oxygen radical absorbance capacity assay; DPPH: 2,2-diphenyl-1-picrylhydrazyl radical (DPPH) scavenging activity assay; FRAP: ferric reducing antioxidant power assay; FICA: ferreous iron chelating activity assay; TE: trolox equivalents; EDTAE: ethylenediaminetetraacetic acid equivalents; DW: extract dry weight; WPE-20: whole-plant extract from material collected in 2020; APE-20: aerial-part extract from material collected in 2020; LE-20: leaf extract from material collected in 2020; FE-20: flower extract from material collected in 2020; SE-20: stem extract from material collected in 2020; RE-20: root extract from material collected in 2020.

**Table 6:** In vitro chemical antioxidant activity of the ethanol extracts from *Impatiens hawkeri* leaves dried by different techniques.

Extract	ORAC (mmol TE/g DW)	DPPH (mmol TE/g DW)	PFRAP (mmol TE/g DW)	FICA (µmol EDTAE/g DW)
FL-21	7.15 ± 0.02 ^A^	2.32 ± 0.13 ^A^	1.88 ± 0.05 ^A^	16.83 ± 0.24 ^A^
OL-21	6.73 ± 0.10 ^A^	1.35 ± 0.11 ^B^	1.03 ± 0.03 ^B^	32.16 ± 1.12 ^B^
SL-21	4.21 ± 0.36 ^B^	1.17 ± 0.07 ^B^	0.79 ± 0.03 ^C^	34.41 ± 0.43 ^C^
Control (quercetin)	23.11 ± 0.83 ^C^	5.87 ± 0.75 ^C^	12.53 ± 1.06 ^D^	23.32 ± 0.83 ^D^

All values are mean ± SD of three replicates. Different superscript letters in the same column indicate significant differences (*p* < 0.05). ORAC: oxygen radical absorbance capacity assay; DPPH: 2,2-diphenyl-1-picrylhydrazyl radical (DPPH) scavenging activity assay; FRAP: ferric reducing antioxidant power assay; FICA: ferreous iron chelating activity assay; TE: trolox equivalents; EDTAE: ethylenediaminetetraacetic acid equivalents; DW: extract dry weight; FL-21: freeze-dried leaf extract from material collected in 2021; OL-21: oven-dried leaf extract from material collected in 2021; SL-21: shade-dried leaf extract from material collected in 2021.

**Table 7:** In vitro antibacterial activity of the ethanol extracts from different plant parts of *Impatiens hawkeri*.

Extract	Minimal Inhibitory Concentration (MIC) (mg/mL)							
	S.A	S.E	E.F	E.C	K.P	P.A	S.E.T	AAI
WPE-20	>10	>10	>10	>10	>10	>10	>10	0
APE-20	>10	>10	>10	>10	>10	>10	>10	0
LE-20	10.00 ± 0.00 ^A^	8.33 ± 2.89 ^A^	10.00 ± 0.00 ^A^	>10	>10	>10	>10	0.32
FE-20	8.33 ± 2.89 ^A^	10.00 ± 0.00 ^A^	>10	>10	>10	>10	>10	0.22
SE-20	>10	>10	>10	>10	>10	>10	>10	0
RE-20	>10	>10	>10	>10	>10	>10	>10	0
Control *	2.75 ± 0.64 ^B^	2.00 ± 0.00 ^B^	0.50 ± 0.00 ^B^	0.03 ± 0.00	0.03 ± 0.00	24.00 ± 6.19	0.09 ± 0.02	N.D

All values are mean ± SD of three replicates. * Control: ciprofloxacin chloride for *E. coli* and *E. faecalis*, or ceftriaxone disodium salt for other bacteria. Control MICs are expressed in µg/mL. Different superscript letters in the same column indicate significant differences (*p* < 0.05). S.A: *Staphylococcus aureus*; S.E: *Staphylococcus epidermidis*; E.F: *Enterococcus faecalis*; E.C: *Escherichia coli*; K.P: *Klebsiella pneumoniae*; P.A: *Pseudomonas aeruginosa*; S.E.T: *Salmonella enterica* subsp. *enterica* serovar Typhimurium; AAI: antibacterial activity index; N.D: not determined; WPE-20: whole-plant extract from material collected in 2020; APE-20: aerial-part extract from material collected in 2020; LE-20: leaf extract from material collected in 2020; FE-20: flower extract from material collected in 2020; SE-20: stem extract from material collected in 2020; RE-20: root extract from material collected in 2020.

**Table 8:** In vitro antibacterial activity of the ethanol extracts from *Impatiens hawkeri* leaves dried by different techniques.

Extract	Minimal Inhibitory Concentration (MIC) (mg/mL)							
	S.A	S.E	E.F	E.C	K.P	P.A	S.E.T	AAI
FL-21	>10	5.00 ± 0.00 ^A^	10.00 ± 0.00 ^A^	>10	>10	>10	>10	0.27
OL-21	5.00 ± 0.00 ^A^	6.67 ± 2.89 ^A^	10.00 ± 0.00 ^A^	>10	>10	>10	>10	0.42
SL-21	>10	5.00 ± 0.00 ^A^	>10	>10	>10	>10	>10	0.20
Control *	2.75 ± 0.64 ^B^	2.00 ± 0.00 ^B^	0.50 ± 0.00 ^B^	0.03 ± 0.00	0.03 ± 0.00	24.00 ± 6.19	0.09 ± 0.02	N.D

All values are mean ± SD of three replicates. * Control: ciprofloxacin chloride for *E. coli* and *E. faecalis*, or ceftriaxone disodium salt for other bacteria. Control MICs are expressed in µg/mL. Different superscript letters in the same column indicate significant differences (*p* < 0.05). S.A: *Staphylococcus aureus*; *Staphylococcus epidermidis*; E.F: *Enterococcus faecalis*; E.C: *Escherichia coli*; K.P: *Klebsiella pneumoniae*; P.A: *Pseudomonas aeruginosa*; S.E.T: *Salmonella enterica* subsp. *enterica* serovar Typhimurium; AAI: antibacterial activity index; N.D: not determined; FL-21: freeze-dried leaf extract from material collected in 2021; OL-21: oven-dried leaf extract from material collected in 2021; SL-21: shade-dried leaf extract from material collected in 2021.

## Data Availability

The original contributions presented in this study are included in the article/Supplementary Material. Further inquiries can be directed to the corresponding author.

## Supplementary Materials

The following supporting information can be downloaded at: https://www.mdpi.com/article/10.3390/plants14071092/s1, Table S1. Weight percentage of fresh material corresponding to each plant part from *Impatiens hawkeri*. Table S2. Loss on drying for investigated *Impatiens hawkeri* plant material. Table S3. Extraction yield for each plant material from *Impatiens hawkeri*. Table S4. Analytical parameters for high-performance thin-layer chromatography methods (HPTLC). Figure S1. Absorbance densitograms for the determination of quercetin (1) and kaempferol (2) obtained at 380 nm after development of the chromatography plate with mobile phase 1 (toluene–ethyl acetate–formic acid (60:45:3 *v*/*v*/*v*)). Figure S2. Absorbance densitograms for the determination of quercetin (1) and kaempferol (2) obtained at 380 nm after consecutive development of the chromatography plate with mobile phases 1 (toluene–ethyl acetate–formic acid (60:45:3 *v*/*v*/*v*)) and 2 (toluene–ethyl acetate–n-hexane: formic acid (60:30:10:3 *v*/*v*/*v*/*v*)). Figure S3. Absorbance densitograms for the determination of rutin (1) and isoquercetin (2) obtained at 365 nm after development of the chromatography plate with mobile phase 3 (butan-2-ol–*n*-butanol–ethyl acetate–formic acid (60:40:15:10 *v*/*v*/*v*/*v*)). Figure S4. Absorbance densitograms for the determination of lawsone (1) and 2-methoxy-1,4-naphtoquinone (2-MNQ) (2) obtained at 275 nm after consecutive development of the chromatography plate with mobile phases 4 (toluene–ethyl acetate–acetic acid (80:30:3 *v*/*v*/*v*)) and 5 (toluene–ethyl acetate–*n*-hexane: acetic acid (80:30:20:10 *v*/*v*/*v*/*v*)). Figure S5. Fluorescence densitograms for the determination of scopoletin (1) obtained at an excitation wavelength of 302 nm and by reading the fluorescence intensity with a K400 detection filter after consecutive development of the chromatography plate with mobile phases 6 (chloroform–ethyl acetate–formic acid (60:30:10 *v*/*v*/*v*)) and 7 (chloroform–ethyl acetate (60:40 *v*/*v*)). Figure S6. In vitro cytotoxicity evaluation of extracts on Vero cells (ATCC CCL-81). Figure S7. Dose-response trends observed with the extracts and quercetin in the intracellular ROS production inhibition assay (IROS) on Vero cells (ATCC CCL-81).
