# Supplementary material for: Influence of Plant Part Selection and Drying Technique: Exploration and Optimization of Antioxidant and Antibacterial Activities of New Guinea Impatiens Extracts
Source: Plants (Basel). 2025 Apr 1;14(7):1092. doi: 10.3390/plants14071092 (PMC11991338; doi:10.3390/plants14071092)
Supplement: Supplementary file 1 [file plants-14-01092-s001.zip › plants-3523055-supplementary.pdf]

# Influence of Plant Part Selection and Drying Technique: Exploration and Optimization of the Antioxidant and Antibacterial Activities of New Guinea Impatiens Extracts

Fabián Delgado Rodríguez [1], *, Gabriela Azofeifa [2], Silvia Quesada [2], Nien Tzu Weng Huang [1], Arlene Loría Gutiérrez [1], María Fernanda Morales Rojas [1]

[1] Instituto de Investigaciones Farmacéuticas (INIFAR), Facultad de Farmacia, Universidad de Costa Rica, 11501-2060, San José, Costa Rica.

[2] Departamento de Bioquímica, Escuela de Medicina, Universidad de Costa Rica, 11501-2060, San José, Costa Rica.

* Corresponding author. Email address: fabian.delgado@misalud.go.cr

## ***Supplementary Material***

### 1. Yield information for *Impatiens hawkeri* plant parts, drying and extraction:

**Table S1**

Weight percentage of fresh material corresponding to each plant part from *Impatiens hawkeri.*

| Anatomical part | Average weight in a fresh plant (g) | Percentage of the average weight of a plant (Percentage of the average weight of aerial parts of a plant) |
|---|---|---|
| **Leaves** | 106 | 33 %<br>(42 %) |
| **Flowers** | 25 | 8 %<br>(10 %) |
| **Stems** | 121 | 37 %<br>(48 %) |
| **Roots** | 73 | 22 %<br>(0 %) |
| ***Aerial parts** | 252 | 78 %<br>(100 %) |
| **Whole plant** | 325 | 100 %<br>(---) |

*Mixture of leaves, flowers, and stems.

**Table S2**

Loss on drying for investigated *Impatiens hawkeri* plant material.

| **Material (Drying technique)** | **Collection date** | **Drying time (hours)** | **Loss on drying (% w/w)** |
|---|---|---|---|
| Whole plant (Oven-drying*) | February 2020 | 72 | 94 |
| Aerial parts (Oven-drying*) | February 2020 | 72 | 93 |
| Leaves (Oven-drying*) | February 2020 | 48 | 91 |
| Flowers (Oven-drying*) | February 2020 | 72 | 94 |
| Stems (Oven-drying*) | February 2020 | 72 | 95 |
| Roots (Oven-drying*) | February 2020 | 48 | 91 |
| Leaves (Freeze-drying**) | February 2021 | 72 | 89 |
| Leaves (Oven-drying*) | February 2021 | 48 | 88 |
| Leaves (Shade-drying***) | February 2021 | 140 | 89 |

*Drying conditions: Temperature: 70 ± 4 °C.

**Drying conditions: Temperature: -50 ± 2 °C, Pressure: 0.100 mBar.

***Drying conditions: Temperature: 24 ± 7 °C.

**Table S3**
Extraction yield for each plant material from *Impatiens hawkeri*.

| Material (Drying technique) | Collection date | Plant material weigh (g) | Extract weight (g) | Yield (% w/w) |
|---|---|---|---|---|
| Whole plant (Oven-drying*) | February 2020 | 50.14 | 3.51 | 7.00 |
| Aerial parts (Oven-drying*) | February 2020 | 50.01 | 3.55 | 7.10 |
| Leaves (Oven-drying*) | February 2020 | 50.00 | 2.42 | 4.84 |
| Flowers (Oven-drying*) | February 2020 | 14.93 | 3.36 | 22.52 |
| Stems (Oven-drying*) | February 2020 | 50.05 | 5.38 | 10.75 |
| Roots (Oven-drying*) | February 2020 | 50,00 | 2.74 | 5.49 |
| Leaves (Freeze-drying**) | February 2021 | 50.30 | 3.68 | 7.32 |
| Leaves (Oven-drying*) | February 2021 | 50.10 | 3.70 | 7.39 |
| Leaves (Shade-drying***) | February 2021 | 50.00 | 4.32 | 8.63 |

*Drying conditions: Temperature: 70 ± 4 °C.
**Drying conditions: Temperature: -50 ± 2 °C, Pressure: 0.100 mBar.
***Drying conditions: Temperature: 24 ± 7 °C.

**2. Analytical information for high-performance thin layer chromatography methods (HPTLC):**

**Table S4**
Analytical parameters for high-performance thin layer chromatography methods (HPTLC).

| **Compound** | **Calibration curve** | **Limit of detection (ng/band)** | **Limit of quantification (ng/band)** | **Recovery ± relative standard deviation (%)** |
|---|---|---|---|---|
| Quercetin[a] | Y= 0.75X + 39.95 ($r^2$ = 0.9946) | 37 | 113 | 99 ± 4 (n* = 4) |
| Quercetin[b] | Y = 0.59X – 20.40 ($r^2$ = 0.9992) | 18 | 54 | 99 ± 4 (n = 4) |
| Kaempferol[a] | Y = 0.73X + 18.40 ($r^2$ = 0.9988) | 17 | 52 | 99 ± 2 (n = 4) |
| Kaempferol[b] | Y = 0.60X – 27.95 ($r^2$ = 0.9984) | 20 | 62 | 100 ± 5 (n = 4) |
| Rutin | Y = 0.36X – 24.60 ($r^2$ = 0.9980) | 46 | 140 | 99 ± 4 (n = 6) |
| Isoquercetin | Y = 0,27X – 27.26 ($r^2$ = 0.9984) | 41 | 127 | 99 ± 4 (n = 6) |
| Lawsone | Y = 0.56X – 15.08 ($r^2$ = 0.9997) | 7 | 22 | 98 ± 3 (n = 4) |
| 2-MNQ | Y =0.51X + 79.40 ($r^2$ = 0.9994) | 17 | 50 | 98 ± 3 (n = 4) |
| Scopoletin | Y = 1.78X + 6.56 ($r^2$ = 0.9996) | 10 | 30 | 101 ± 3 (n = 4) |

[a] Method for the analysis of flower extract from material collected in 2020 (FE-20). [b] Method for the analysis of all extracts except for FE-20. *Number of experimental repetitions. 2-MNQ: 2-methoxy-1,4-naphtoquinone.

**3. Sample densitograms for reference standards and extracts obtained during high-performance thin layer chromatography (HPTLC) analyses.**

Abbreviations: WPE-20: Whole plant extract from material collected in 2020; APE-20: Aerial part extract from material collected in 2020; LE-20: Leaf extract from material collected in 2020; FE-20: Flower extract from material collected in 2020; SE-20: Stem extract from material collected in 2020; RE-20: Root extract from material collected in 2020; FL-21: Freeze-dried leaf extract from material collected in 2021; OL-21: Oven-dried leaf extract from material collected in 2021; SL-21: Shade-dried leaf extract from material collected in 2021.

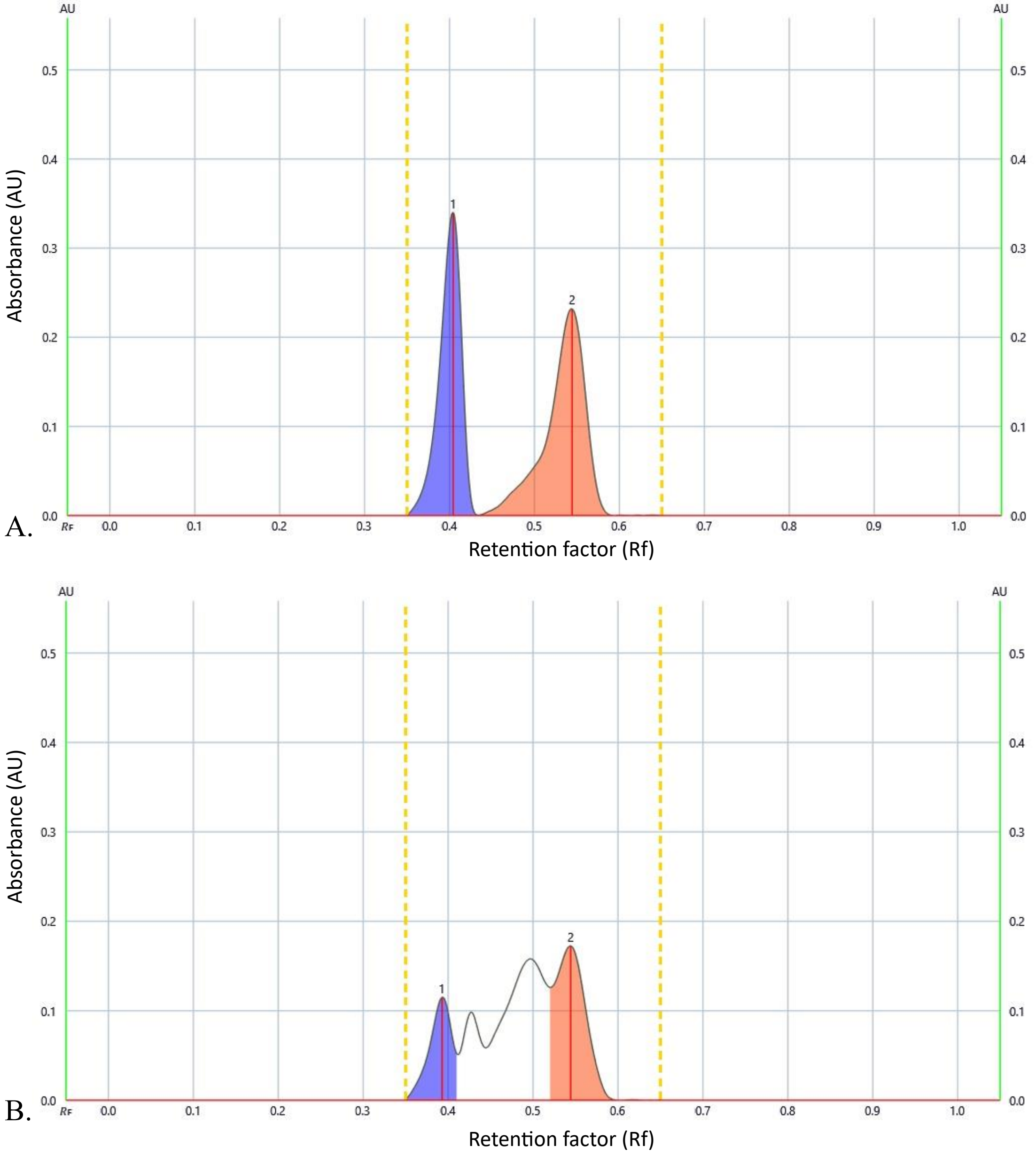


**Figure S1.** Absorbance densitograms for the determination of quercetin (1) and kaempferol (2) obtained at 380 nm after development of the chromatography plate with mobile phase 1 (toluene: ethyl acetate: formic acid (60:45:3 v/v/v)). A. Densitogram obtained with the

application of 1.5 µL of quercetin (240 µg/mL) and kaempferol (200 µg/mL) standard solution. B. Densitogram obtained with the application of 100 µL of FE-20 test solution (10 mg/mL).

A.
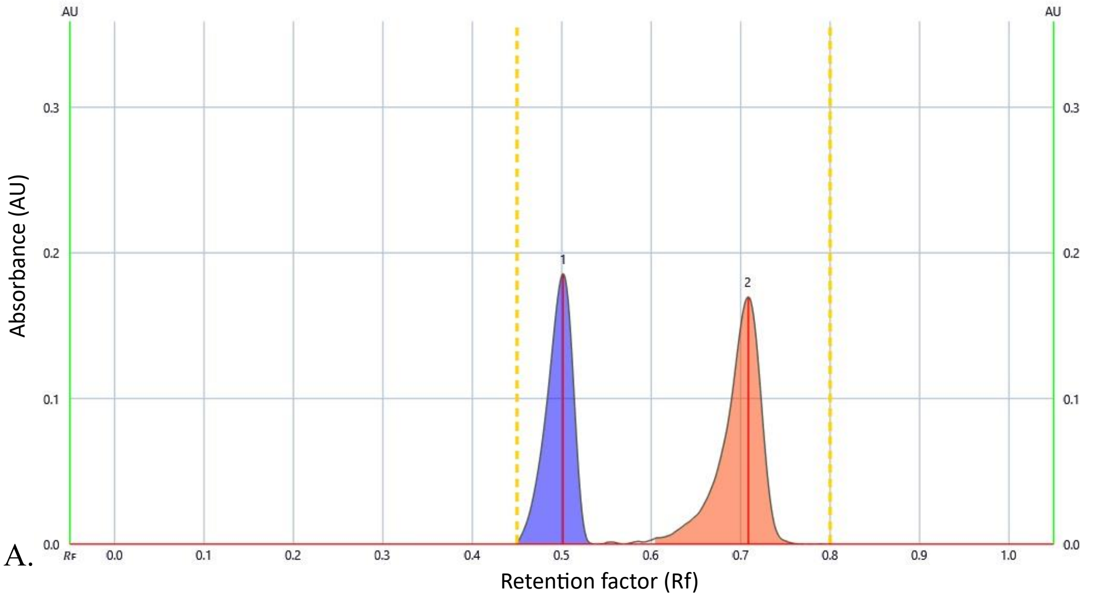


B.
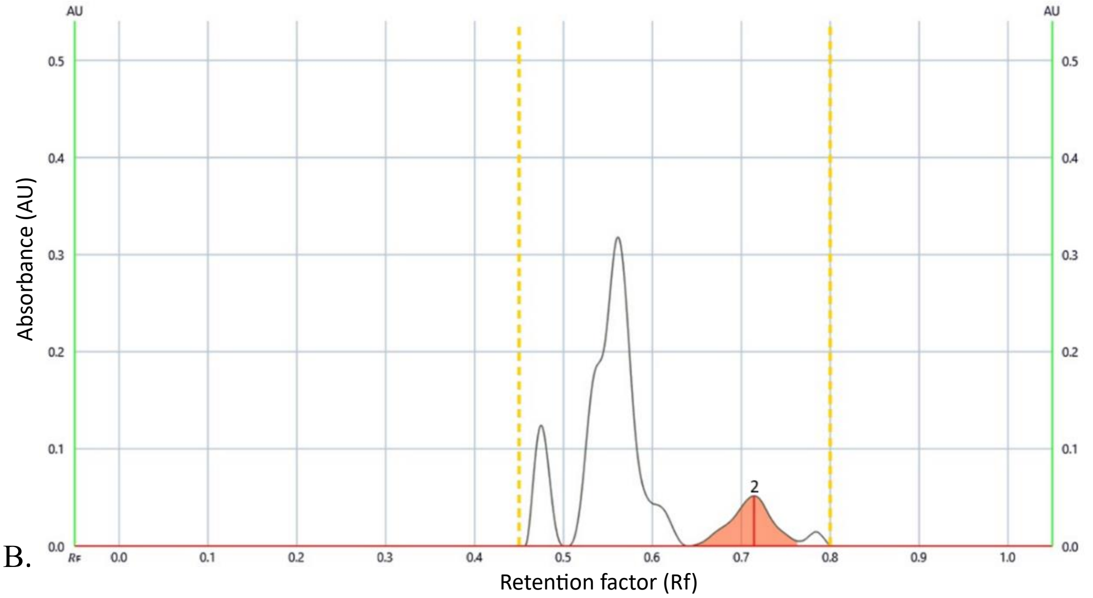


**Figure S2.** Absorbance densitograms for the determination of quercetin (1) and kaempferol (2) obtained at 380 nm after consecutive development of the chromatography plate with mobile phases 1 (toluene: ethyl acetate: formic acid (60:45:3 v/v/v)) and 2 (toluene: ethyl acetate: *n*-hexane: formic acid (60:30:10:3 v/v/v/v)). A. Densitogram obtained with the application of 1.5 µL of quercetin (240 µg/mL) and kaempferol (200 µg/mL) standard solution. B. Densitogram obtained with the application of 50 µL of WPE-20 test solution (10 mg/mL).

C.
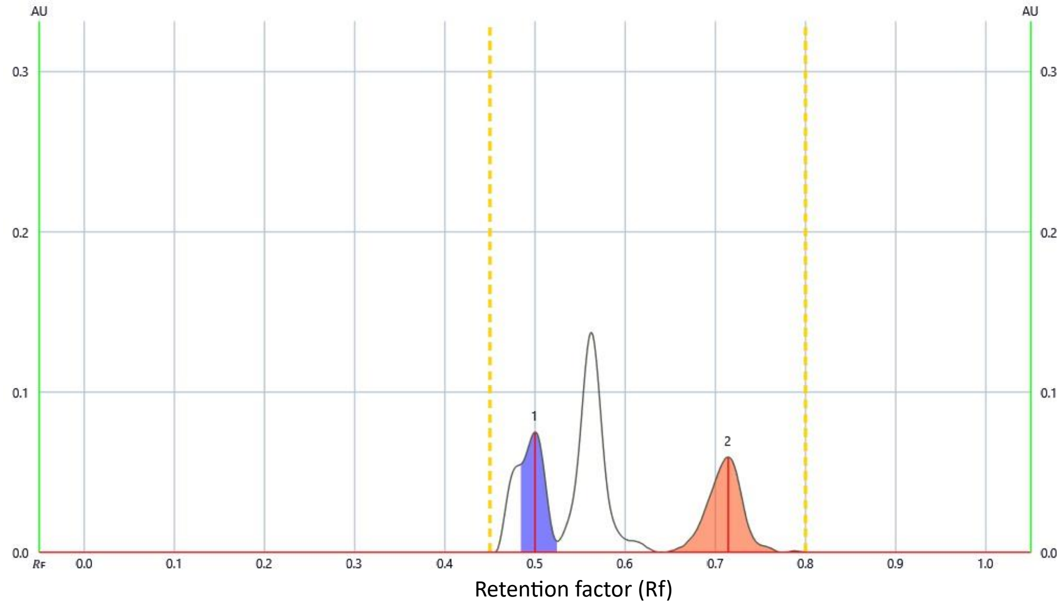


D.
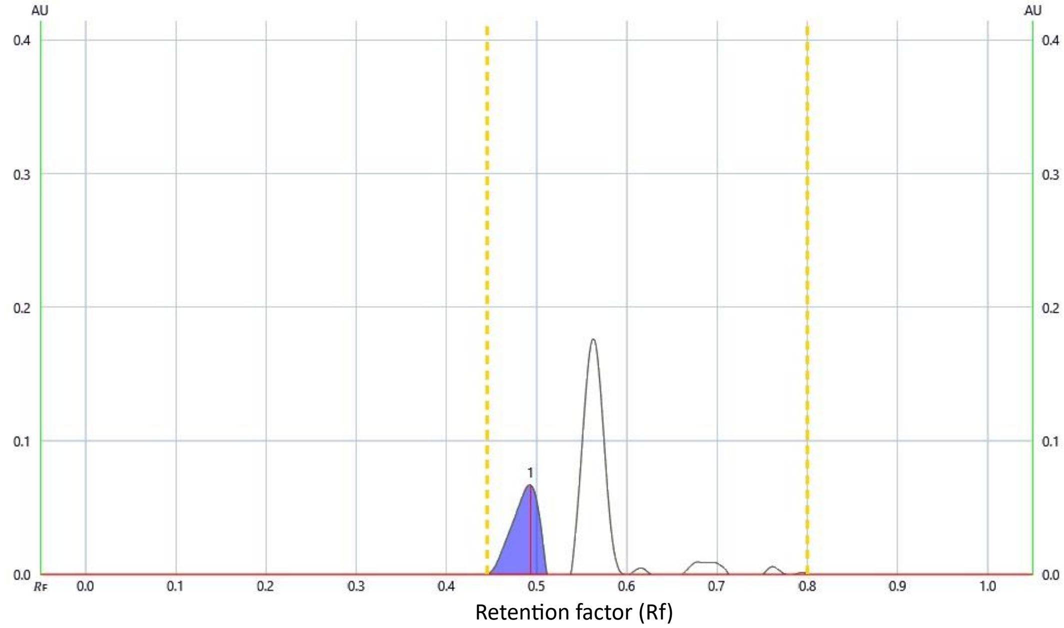


**Figure S2 (continued).** Absorbance densitograms for the determination of quercetin (1) and kaempferol (2) obtained at 380 nm after consecutive development of the chromatography plate with mobile phases 1 (toluene: ethyl acetate: formic acid (60:45:3 v/v/v)) and 2 (toluene: ethyl acetate: *n*-hexane: formic acid (60:30:10:3 v/v/v/v)). C. Densitogram obtained with the application of 50 µL of APE-20 test solution (10 mg/mL). D. Densitogram obtained with the application of 100 µL of LE-20 test solution (10 mg/mL).

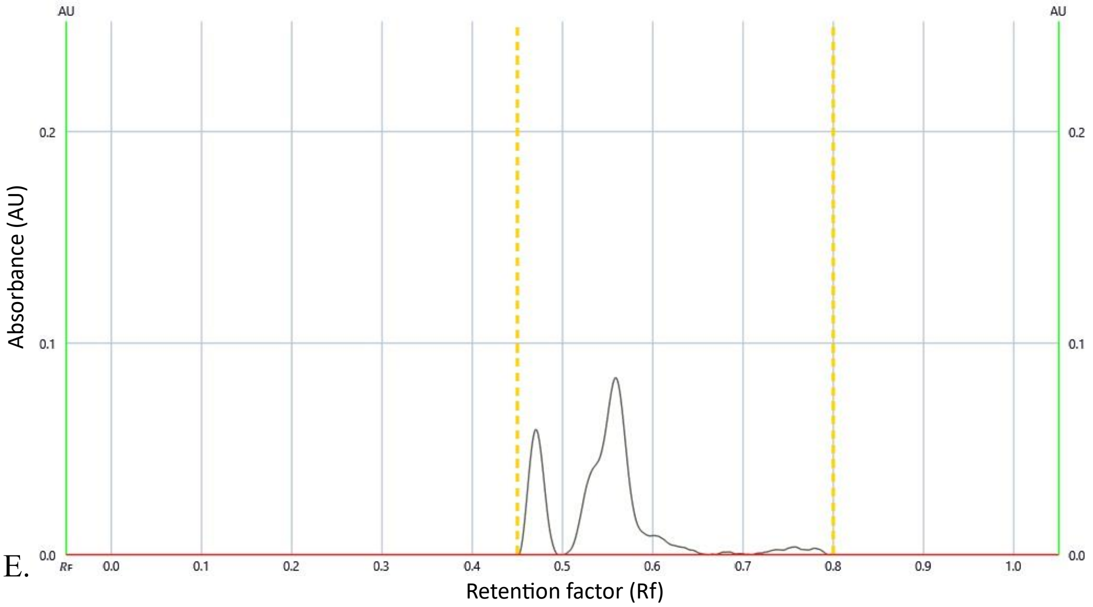


E.

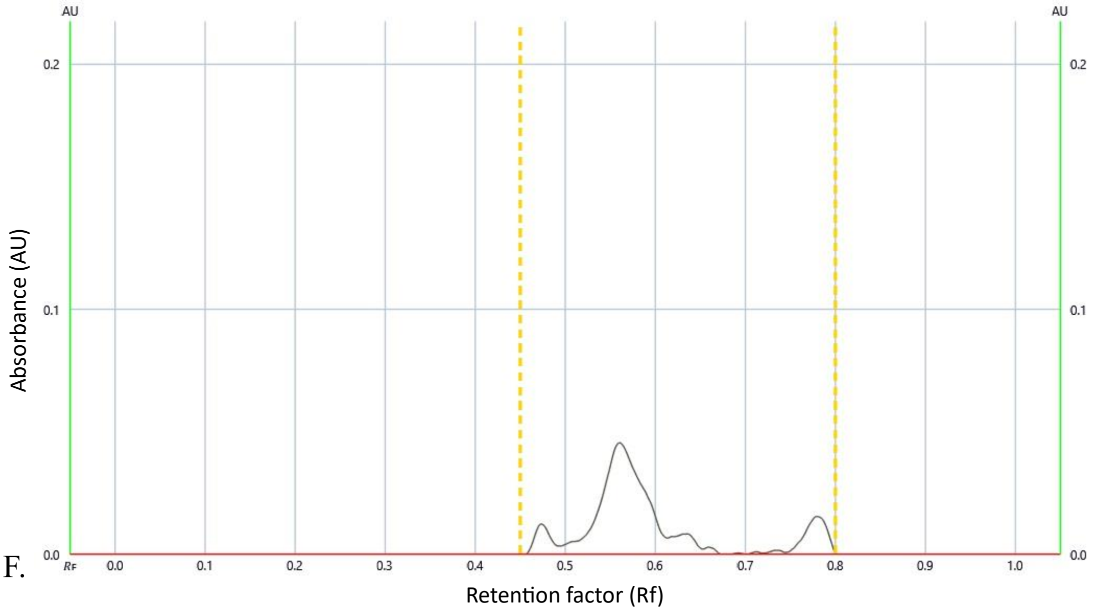


F.

**Figure S2 (continued).** Absorbance densitograms for the determination of quercetin (1) and kaempferol (2) obtained at 380 nm after consecutive development of the chromatography plate with mobile phases 1 (toluene: ethyl acetate: formic acid (60:45:3 v/v/v)) and 2 (toluene: ethyl acetate: *n*-hexane: formic acid (60:30:10:3 v/v/v/v)). E. Densitogram obtained with the application of 40 µL of SE-20 test solution (10 mg/mL). F. Densitogram obtained with the application of 60 µL of RE-20 test solution (10 mg/mL).

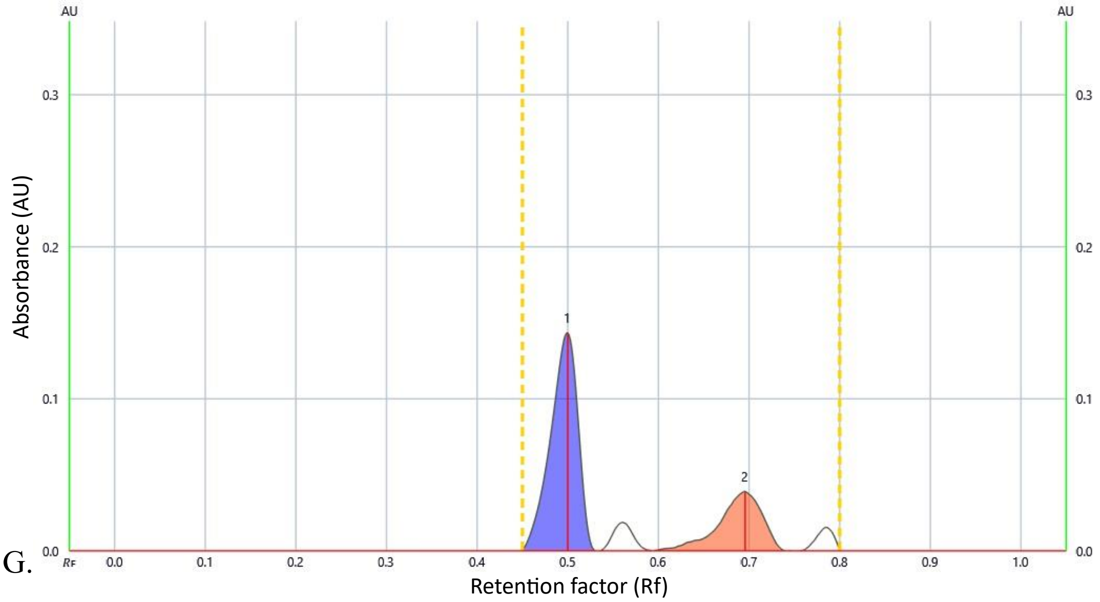


G.

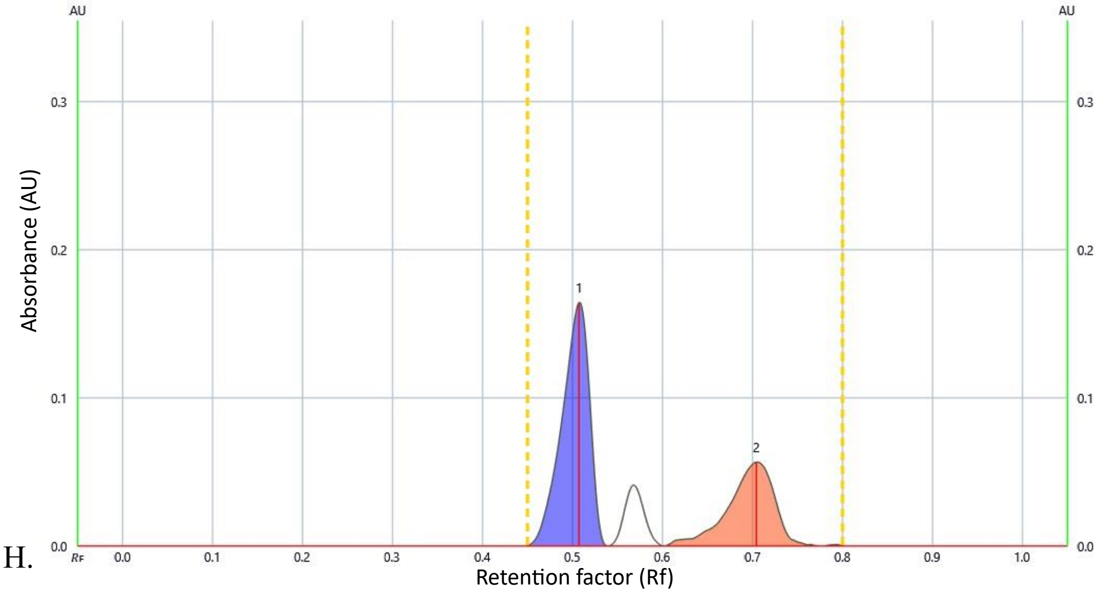


H.

**Figure S2 (continued).** Absorbance densitograms for the determination of quercetin (1) and kaempferol (2) obtained at 380 nm after consecutive development of the chromatography plate with mobile phases 1 (toluene: ethyl acetate: formic acid (60:45:3 v/v/v)) and 2 (toluene: ethyl acetate: *n*-hexane: formic acid (60:30:10:3 v/v/v/v)). G. Densitogram obtained with the application of 50 µL of FL-21 test solution (10 mg/mL). H. Densitogram obtained with the application of 15 µL of OL-21 test solution (10 mg/mL).

I.

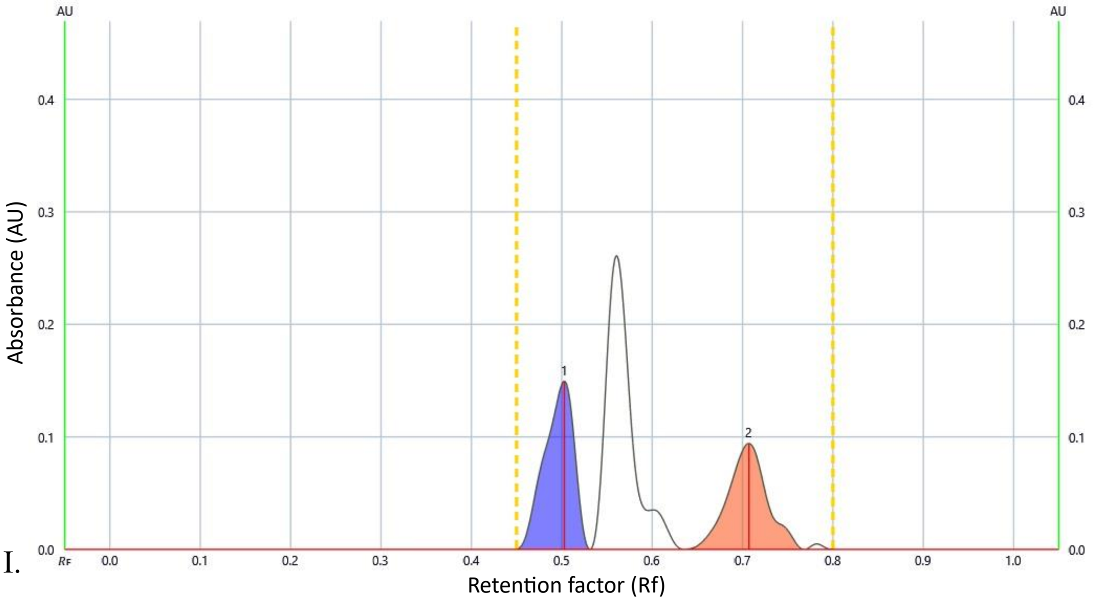


**Figure S2 (continued).** Absorbance densitograms for the determination of quercetin (1) and kaempferol (2) obtained at 380 nm after consecutive development of the chromatography plate with mobile phases 1 (toluene: ethyl acetate: formic acid (60:45:3 v/v/v)) and 2 (toluene: ethyl acetate: *n*-hexane: formic acid (60:30:10:3 v/v/v/v)). I. Densitogram obtained with the application of 10 µL of SL-21 test solution.

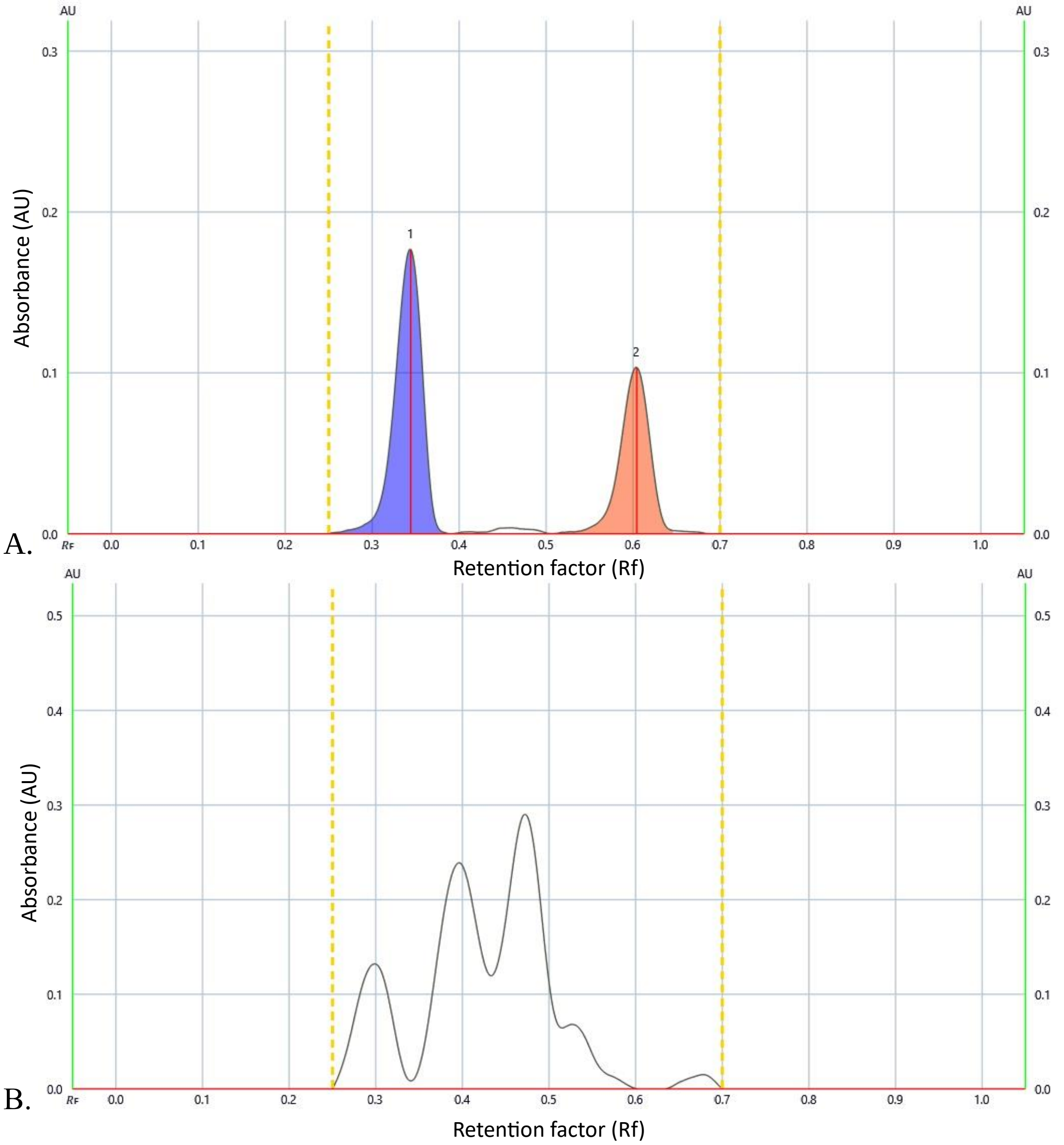


**Figure S3** Absorbance densitograms for the determination of rutin (1) and isoquercetin (2) obtained at 365 nm after development of the chromatography plate with mobile phase 3 (butan-2-ol: *n*-butanol: ethyl acetate: formic acid (60:40:15:10 v/v/v/v)). A. Densitogram obtained with the application of 2.0 µL of rutin (280 µg/mL) and isoquercetin (280 µg/mL) standard solution. B. Densitogram obtained with the application of 25 µL of WPE-20 test solution (10 mg/mL).

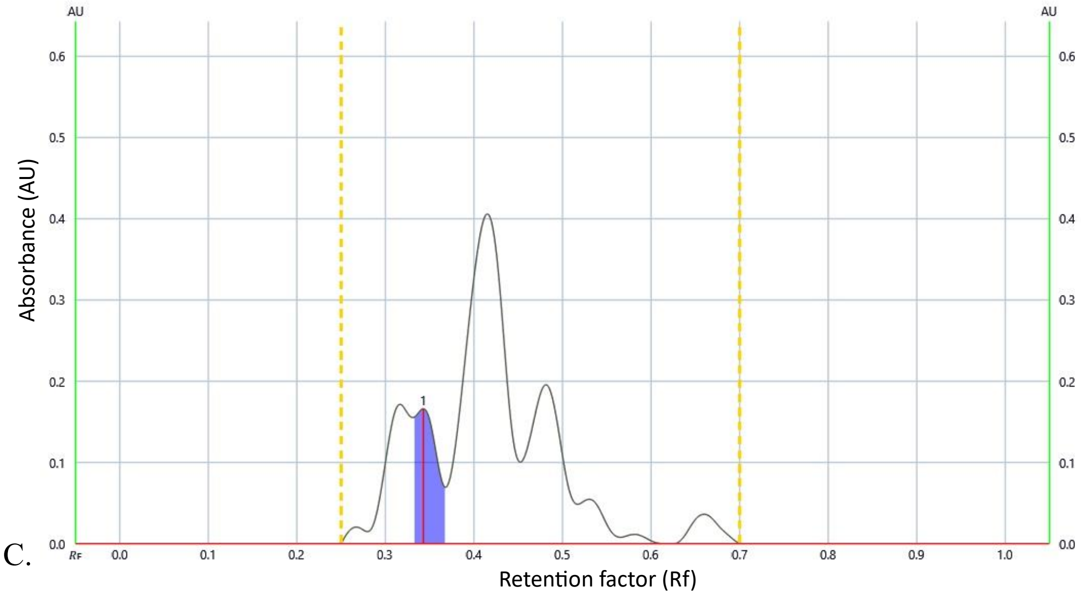


C.

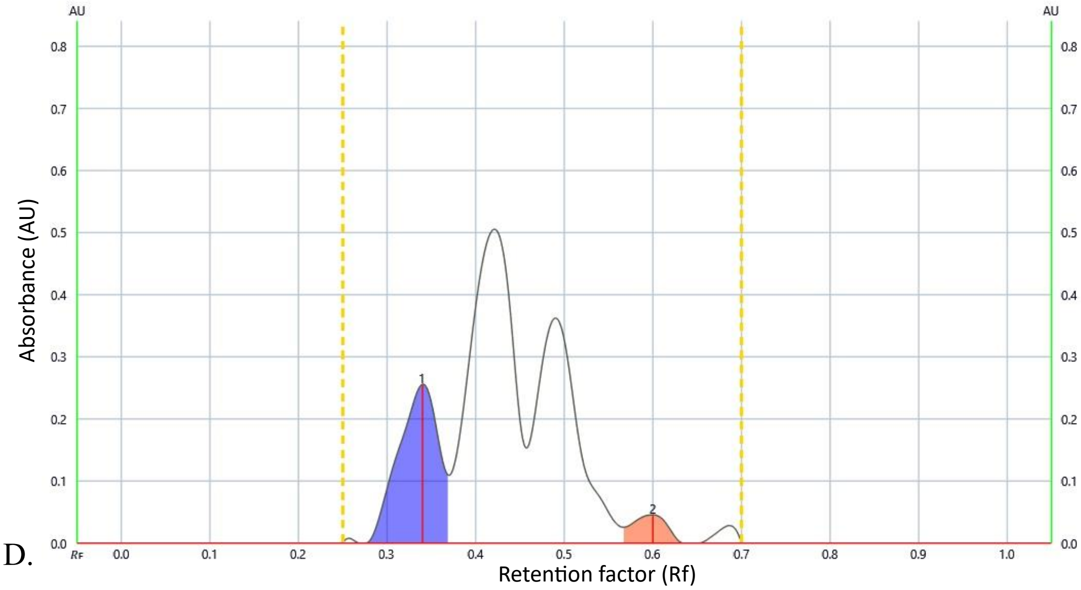


D.

**Figure S3 (continued).** Absorbance densitograms for the determination of rutin (1) and isoquercetin (2) obtained at 365 nm after development of the chromatography plate with mobile phase 3 (butan-2-ol: *n*-butanol: ethyl acetate: formic acid (60:40:15:10 v/v/v/v)). C. Densitogram obtained with the application of 15 µL of APE-20 test solution (10 mg/mL). D. Densitogram obtained with the application of 15 µL of LE-20 test solution (10 mg/mL).

E. 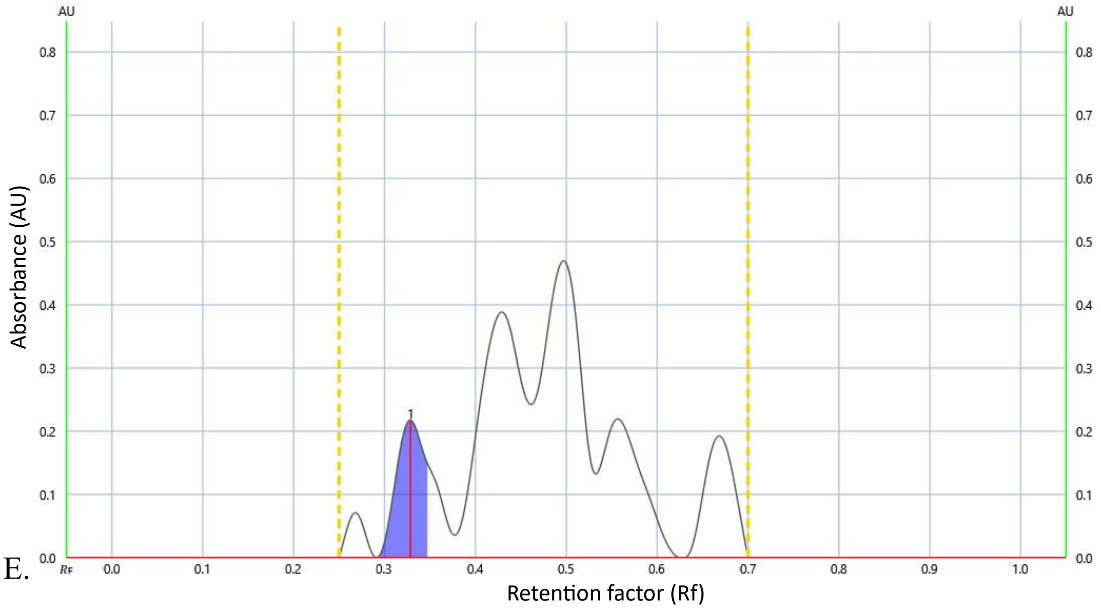


F. 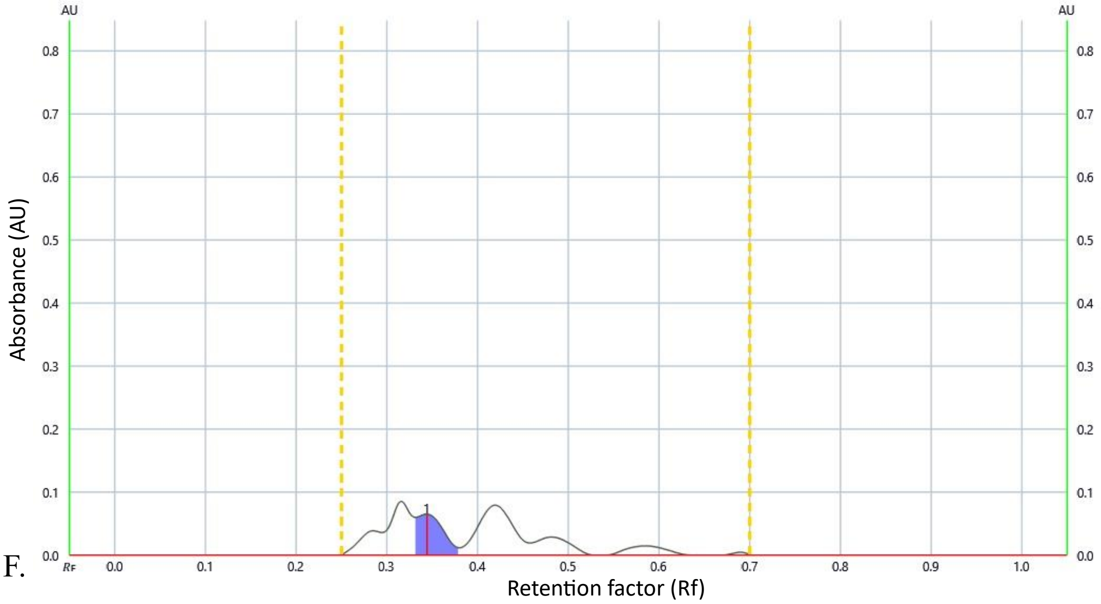


**Figure S3 (continued).** Absorbance densitograms for the determination of rutin (1) and isoquercetin (2) obtained at 365 nm after development of the chromatography plate with mobile phase 3 (butan-2-ol: *n*-butanol: ethyl acetate: formic acid (60:40:15:10 v/v/v/v)). E. Densitogram obtained with the application of 3 µL of FE-20 test solution (10 mg/mL). F. Densitogram obtained with the application of 100 µL of SE-20 test solution (10 mg/mL).

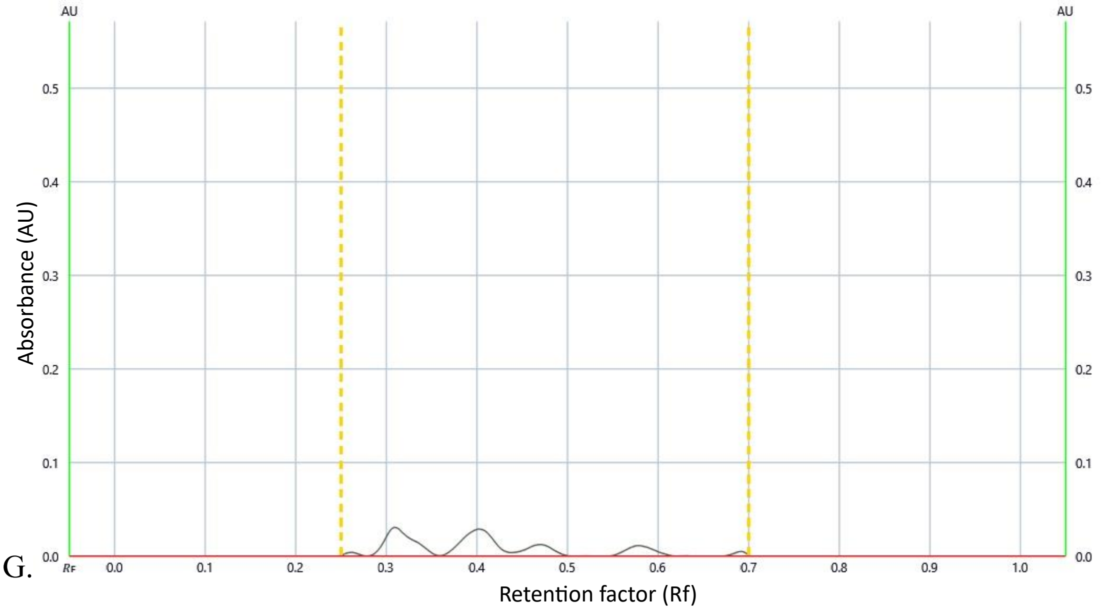


G.

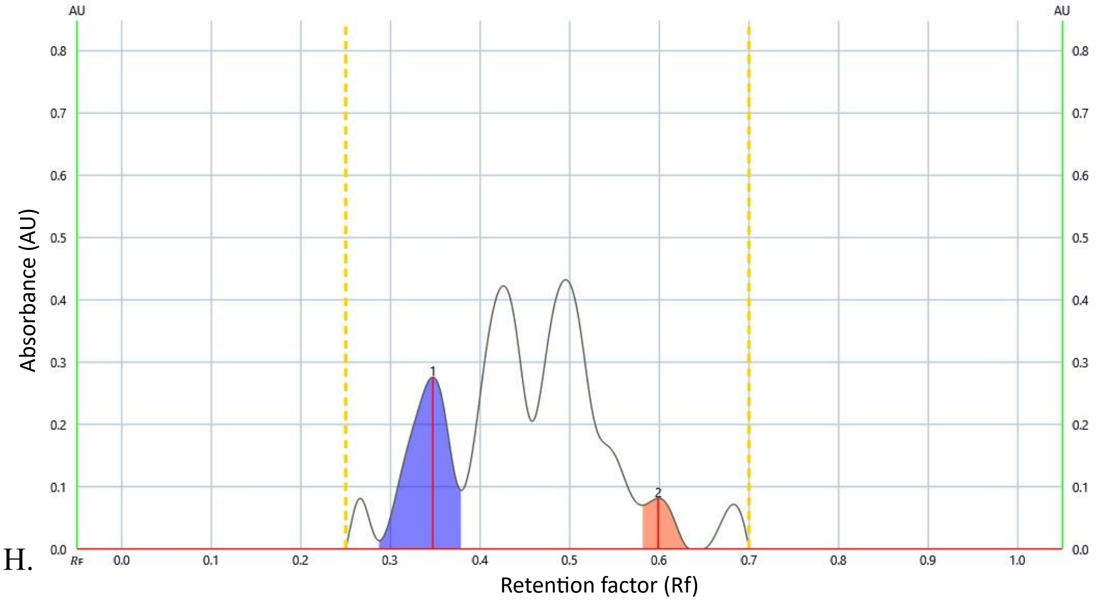


H.

**Figure S3 (continued).** Absorbance densitograms for the determination of rutin (1) and isoquercetin (2) obtained at 365 nm after development of the chromatography plate with mobile phase 3 (butan-2-ol: *n*-butanol: ethyl acetate: formic acid (60:40:15:10 v/v/v/v)). G. Densitogram obtained with the application of 100 µL of RE-20 test solution (10 mg/mL). H. Densitogram obtained with the application of 15 µL of FL-21 test solution (10 mg/mL).

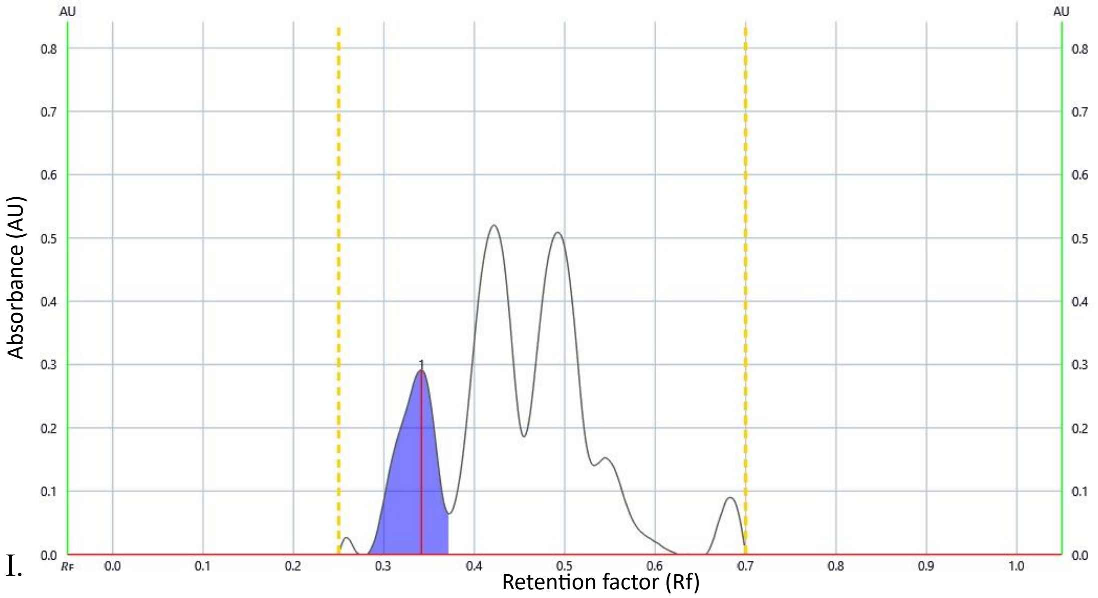


I.

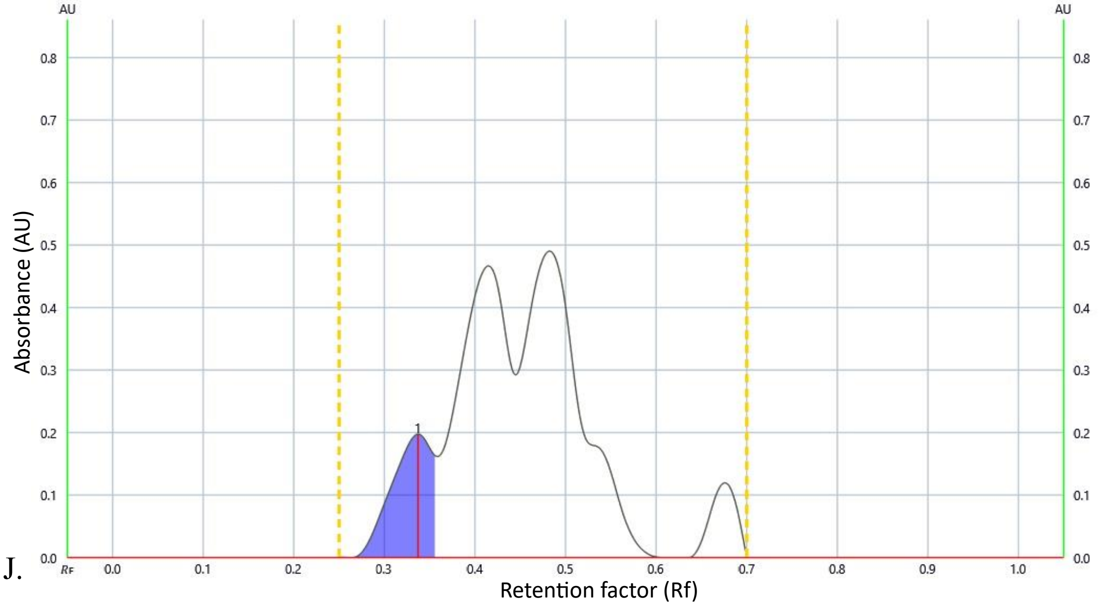


J.

**Figure S3 (continued).** Absorbance densitograms for the determination of rutin (1) and isoquercetin (2) obtained at 365 nm after development of the chromatography plate with mobile phase 3 (butan-2-ol: *n*-butanol: ethyl acetate: formic acid (60:40:15:10 v/v/v/v)). I. Densitogram obtained with the application of 8 µL of the solution of OL-21 test solution (10 mg/mL). J. Densitogram obtained with the application of 20 µL of SL-21 test solution (10 mg/mL).

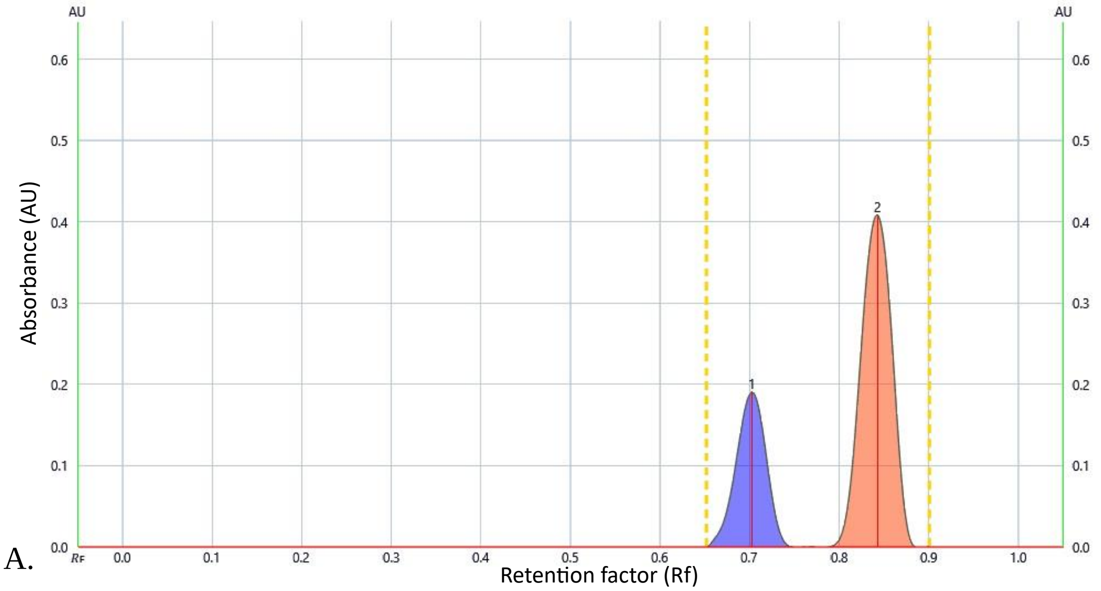


A.

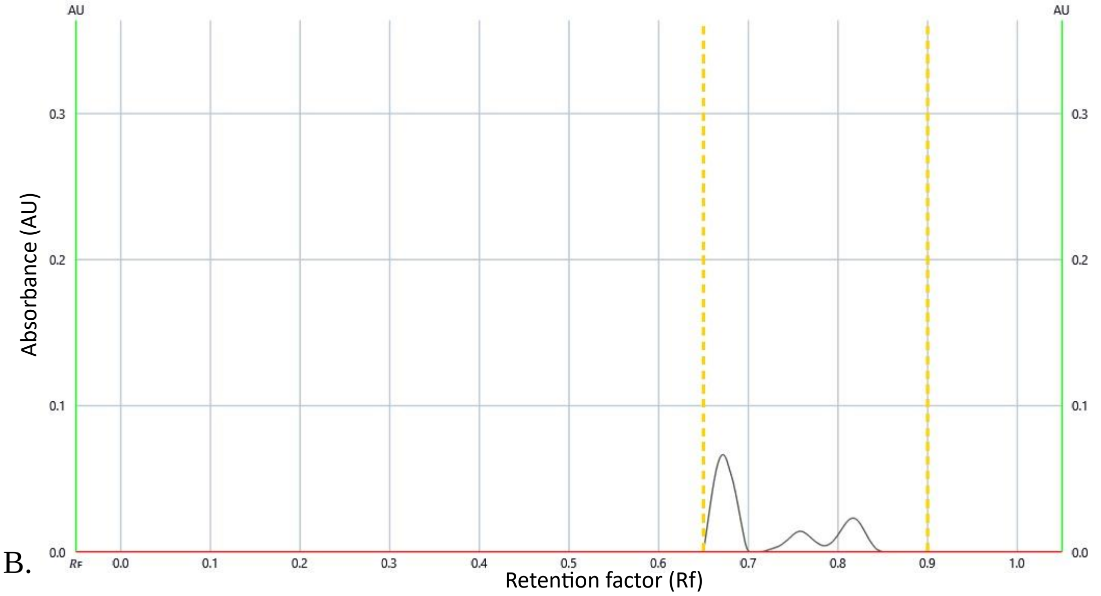


B.

**Figure S4.** Absorbance densitograms for the determination of lawsone (1) and 2-methoxy-1,4-naphtoquinone (2-MNQ) (2) obtained at 275 nm after consecutive development of the chromatography plate with mobile phases 4 (toluene: ethyl acetate: acetic acid (80:30:3 v/v/v)) and 5 (toluene: ethyl acetate: *n*-hexane: acetic acid (80:30:20:10 v/v/v/v)). A. Densitogram obtained with the application of 1.5 µL of lawsone (220 µg/mL) and 2-MNQ (425 µg/mL) standard solution. B. Densitogram obtained with the application of 100 µL of WPE-20 test solution (10 mg/mL).

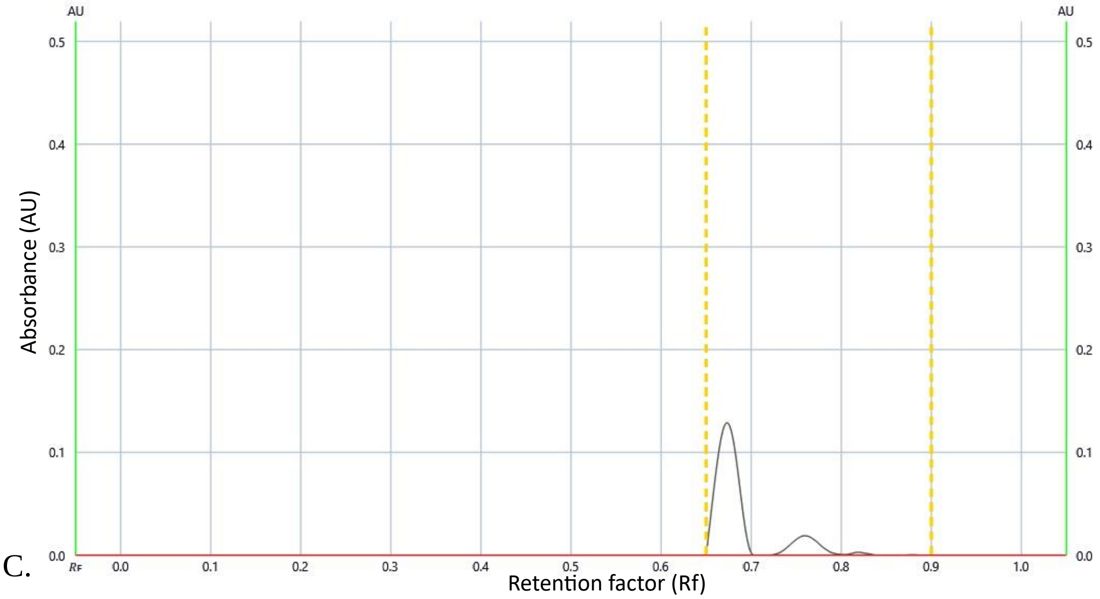


C.

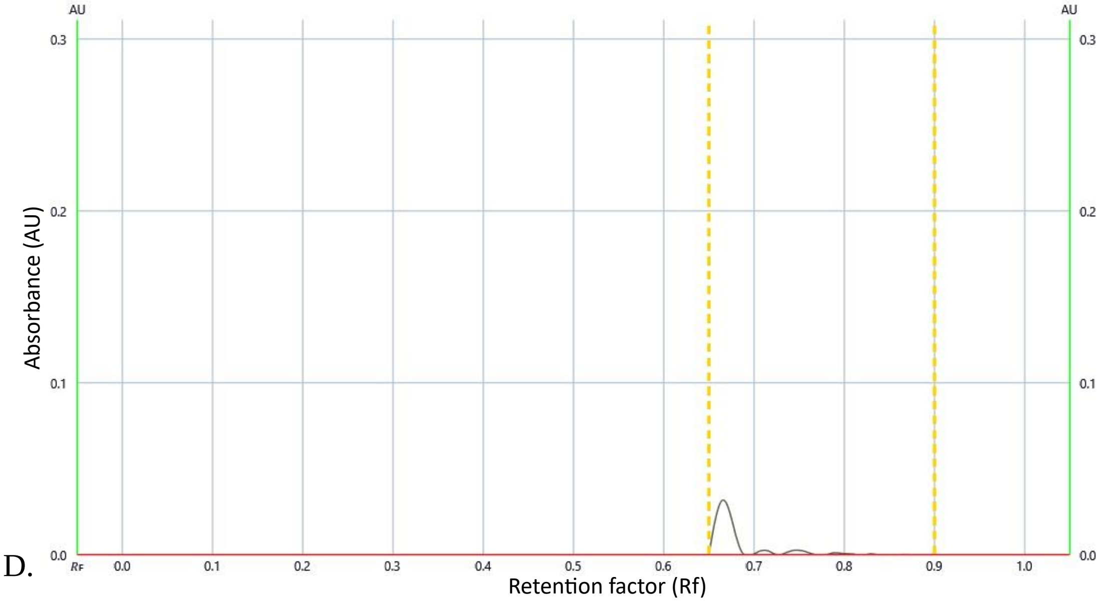


D.

**Figure S4 (continued).** Absorbance densitograms for the determination of lawsone (1) and 2-MNQ (2) obtained at 275 nm after consecutive development of the chromatography plate with mobile phases 4 (toluene: ethyl acetate: acetic acid (80:30:3 v/v/v)) and 5 (toluene: ethyl acetate: *n*-hexane: acetic acid (80:30:20:10 v/v/v/v)). C. Densitogram obtained with the application of 100 µL of APE-20 test solution (10 mg/mL). D. Densitogram obtained with the application of 100 µL of LE-20 test solution (10 mg/mL).

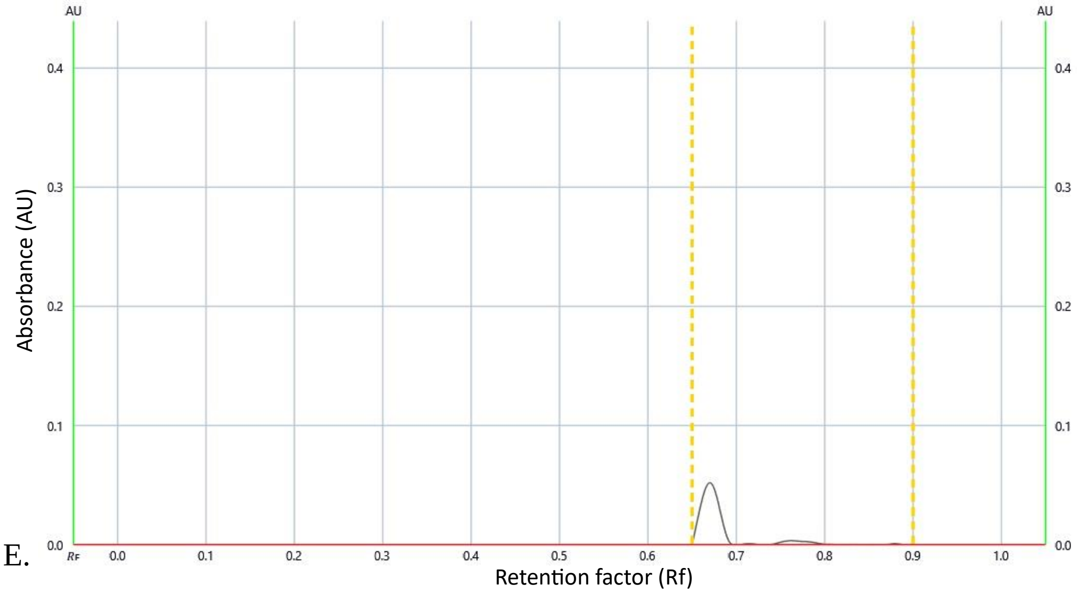


E.

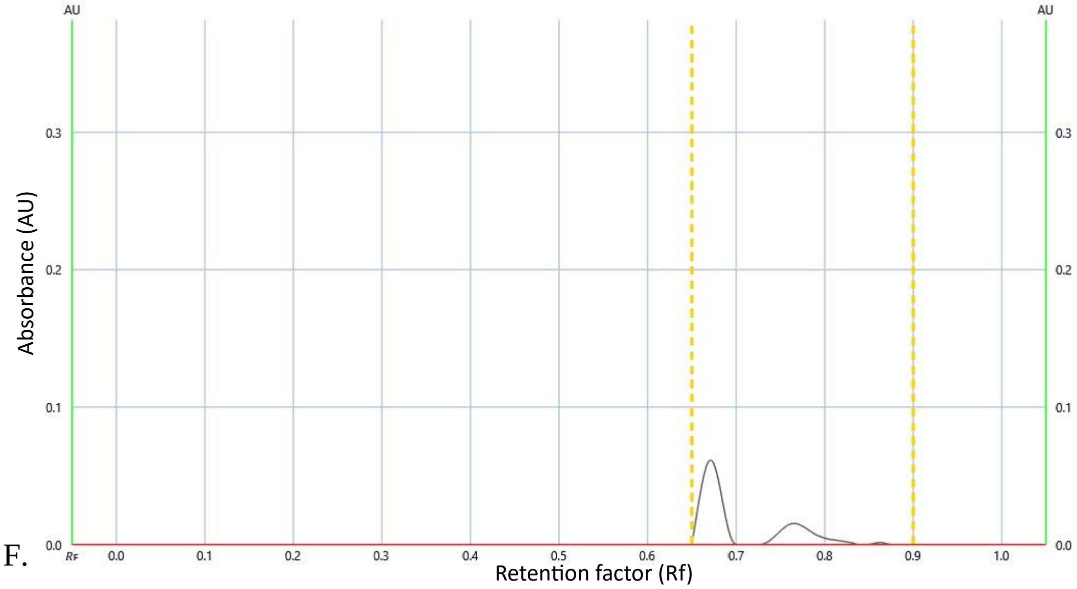


F.

**Figure S4 (continued).** Absorption densitograms for the determination of lawsone (1) and 2-MNQ (2) obtained at 275 nm after consecutive development of the chromatography plate with mobile phases 4 (toluene: ethyl acetate: acetic acid (80:30:3 v/v/v)) and 5 (toluene: ethyl acetate: *n*-hexane: acetic acid (80:30:20:10 v/v/v/v)). E. Densitogram obtained with the application of 100 µL of FE-20 test solution (10 mg/mL). F. Densitogram obtained with the application of 100 µL of SE-20 test solution (10 mg/mL).

G. 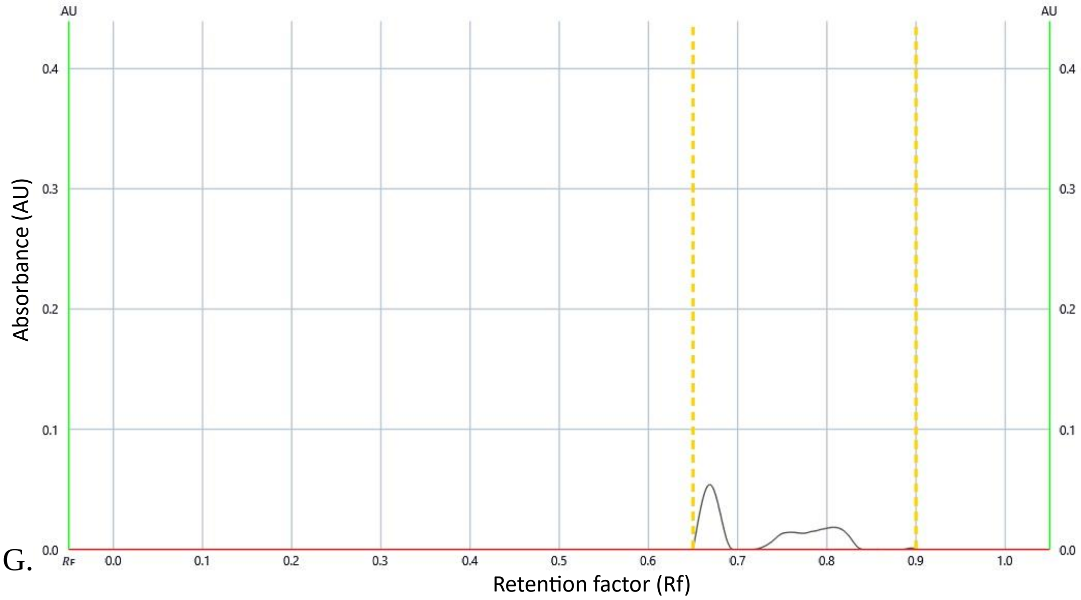


H. 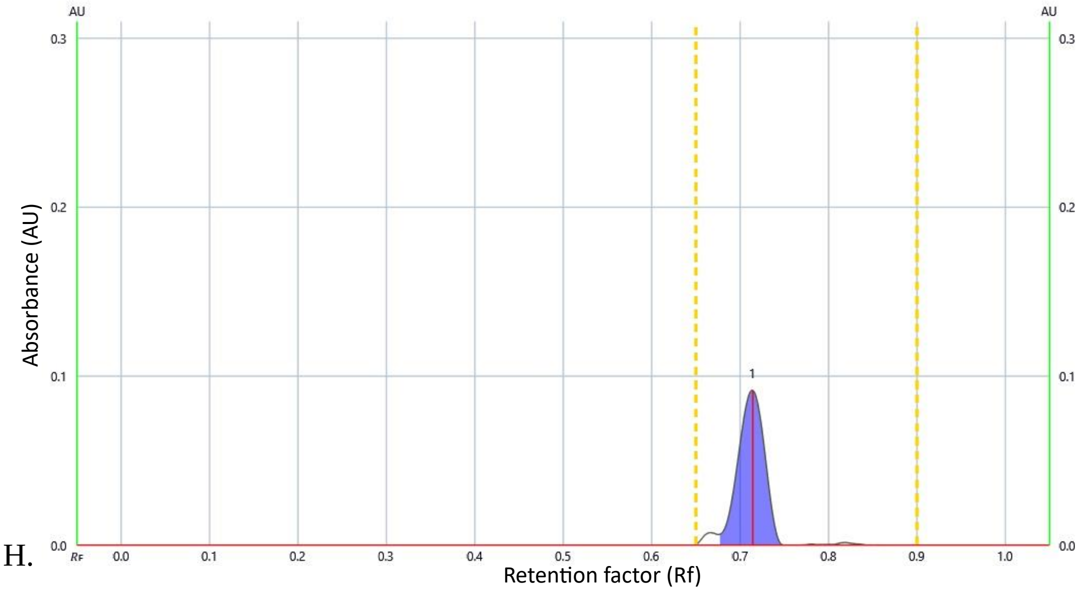


**Figure S4 (continued).** Absorbance densitograms for the determination of lawsone (1) and 2-MNQ (2) obtained at 275 nm after consecutive development of the chromatography plate with mobile phases 4 (toluene: ethyl acetate: acetic acid (80:30:3 v/v/v)) and 5 (toluene: ethyl acetate: *n*-hexane: acetic acid (80:30:20:10 v/v/v/v)). G. Densitogram obtained with the application of 100 µL of RE-20 test solution (10 mg/mL). H. Densitogram obtained with the application of 100 µL of FL-21 test solution (10 mg/mL).

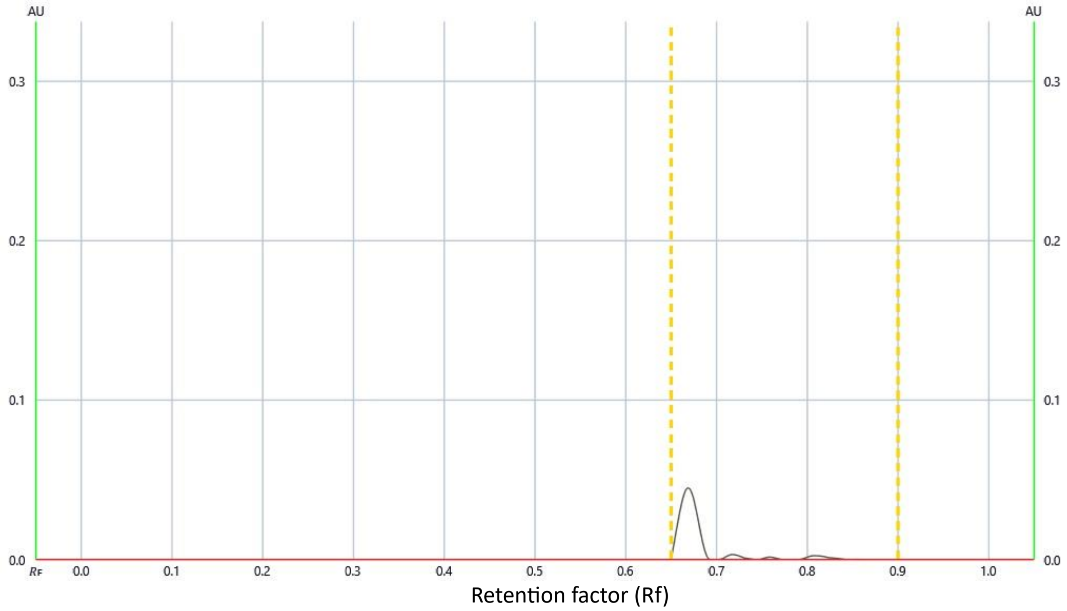


I.

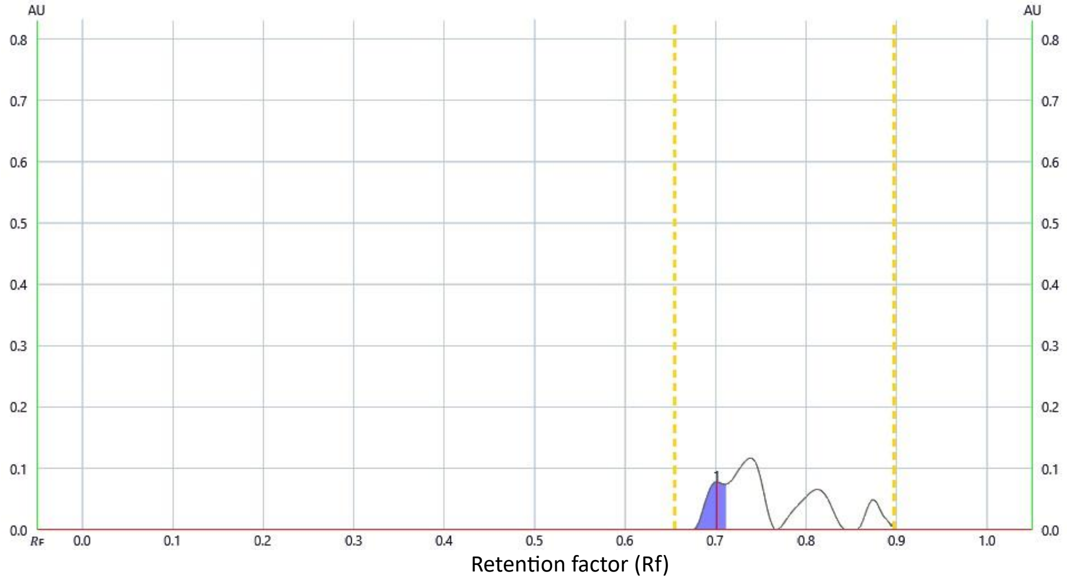


J.

**Figure S4 (continued).** Absorption densitograms for the determination of lawsone (1) and 2-MNQ (2) obtained at 275 nm after consecutive development of the chromatography plate with mobile phases 4 (toluene: ethyl acetate: acetic acid (80:30:3 v/v/v)) and 5 (toluene: ethyl acetate: *n*-hexane: acetic acid (80:30:20:10 v/v/v/v)). I. Densitogram obtained with the application of 100 µL of OL-21 (10 mg/mL). J. Densitogram obtained with the application of 100 µL of SL-21 test solution (10 mg/mL).

A.

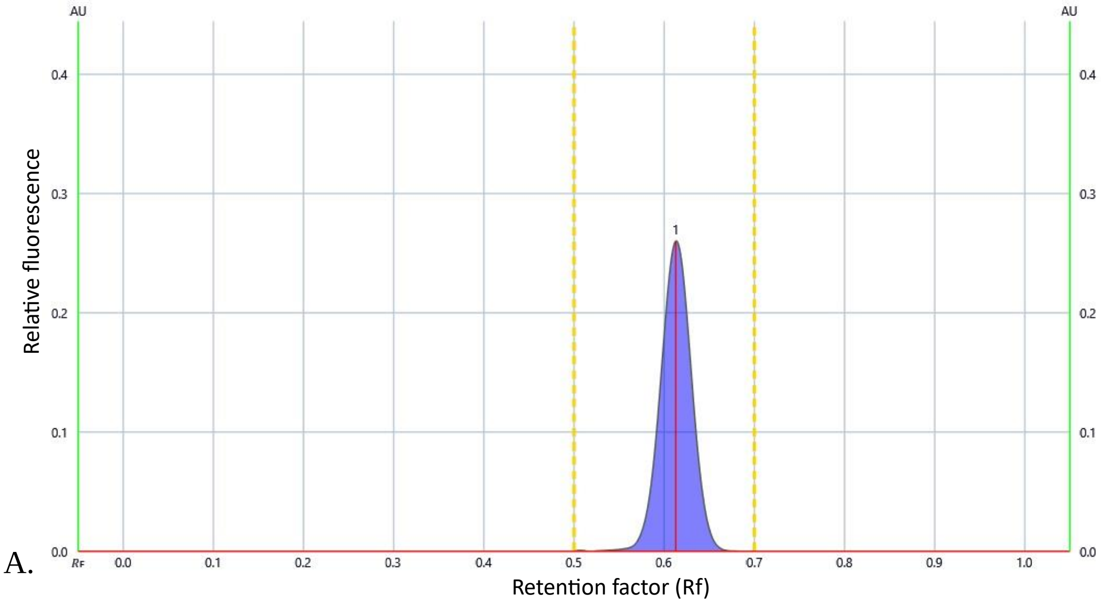


B.

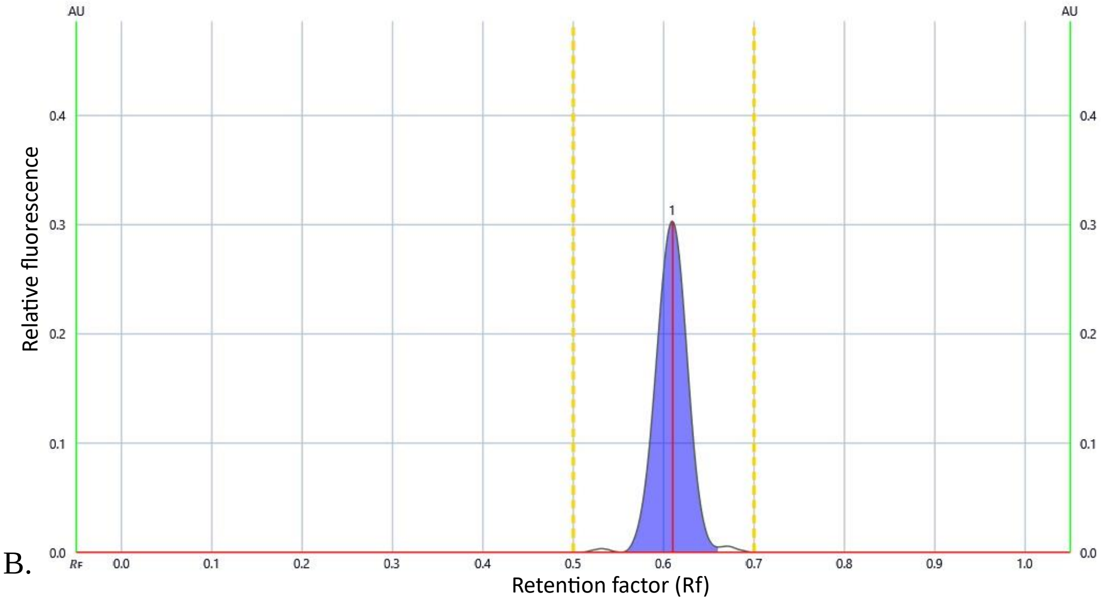


**Figure S5.** Fluorescence densitograms for the determination of scopoletin (1) obtained at an excitation wavelength of 302 nm and by reading the fluorescence intensity with a K400 detection filter after consecutive development of the chromatography plate with mobile phases 6 (chloroform: ethyl acetate: formic acid (60:30:10 v/v/v)) and 7 (chloroform: ethyl acetate (60:40 v/v)). A. Densitogram obtained with the application of 0.5 µL of scopoletin (300 µg/ml) standard solution. B. Densitogram obtained with the application of 25 µL of WPE-20 test solution (10 mg/mL).

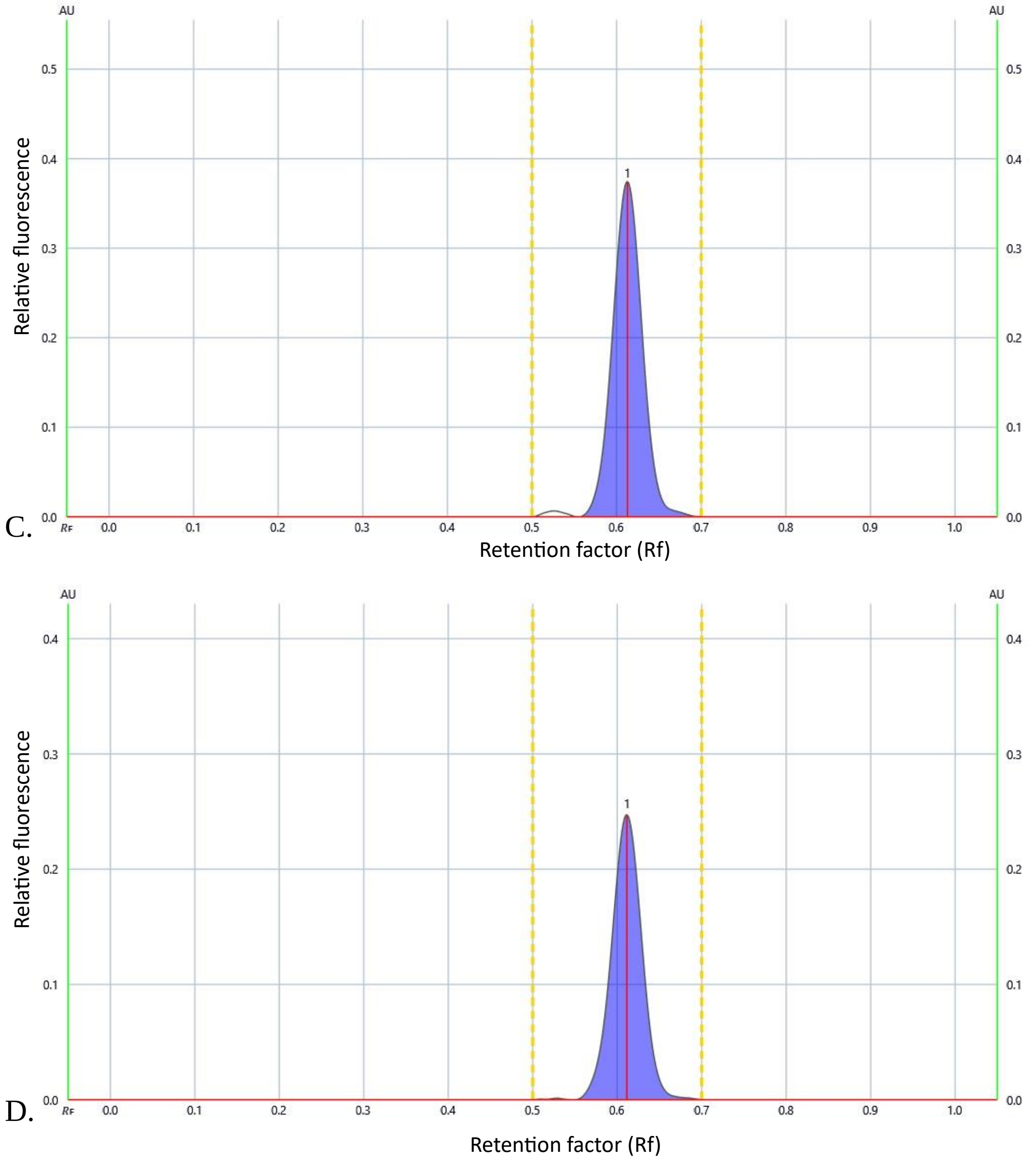


**Figure S5 (continued).** Fluorescence densitograms for the determination of scopoletin (1) obtained at an excitation wavelength of 302 nm and by reading the fluorescence intensity with a K400 detection filter after consecutive development of the chromatography plate with mobile phases 6 (chloroform: ethyl acetate: formic acid (60:30:10 v/v/v)) and 7 (chloroform: ethyl acetate (60:40 v/v)). C. Densitogram obtained with the application of 25 µL of APE-20 test solution (10 mg/mL). D. Densitogram obtained with the application of 10 µL of LE-20 test solution (10 mg/mL).

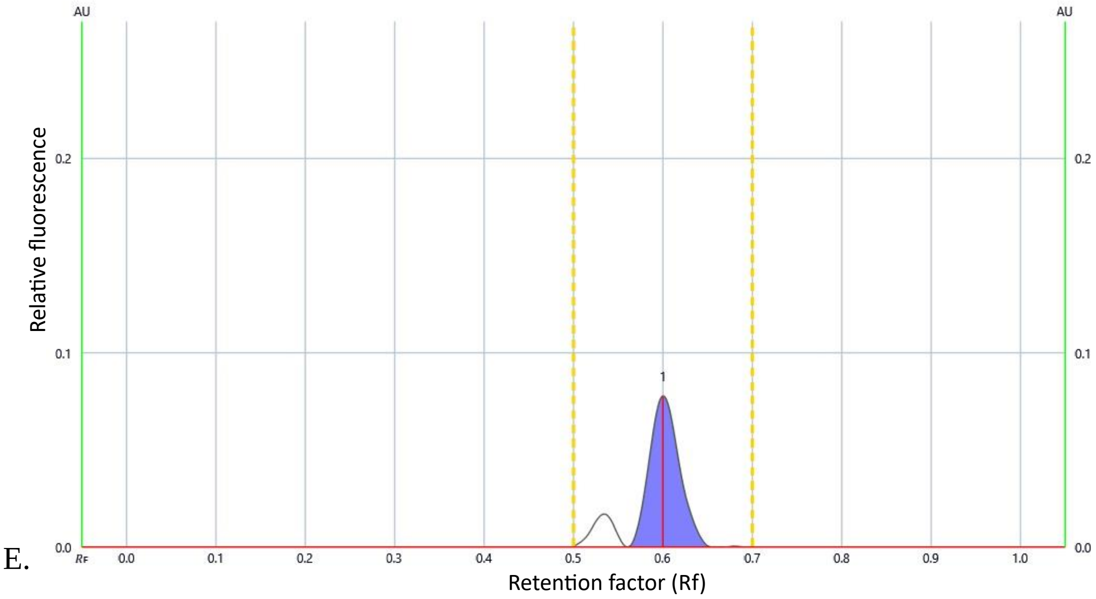


E.

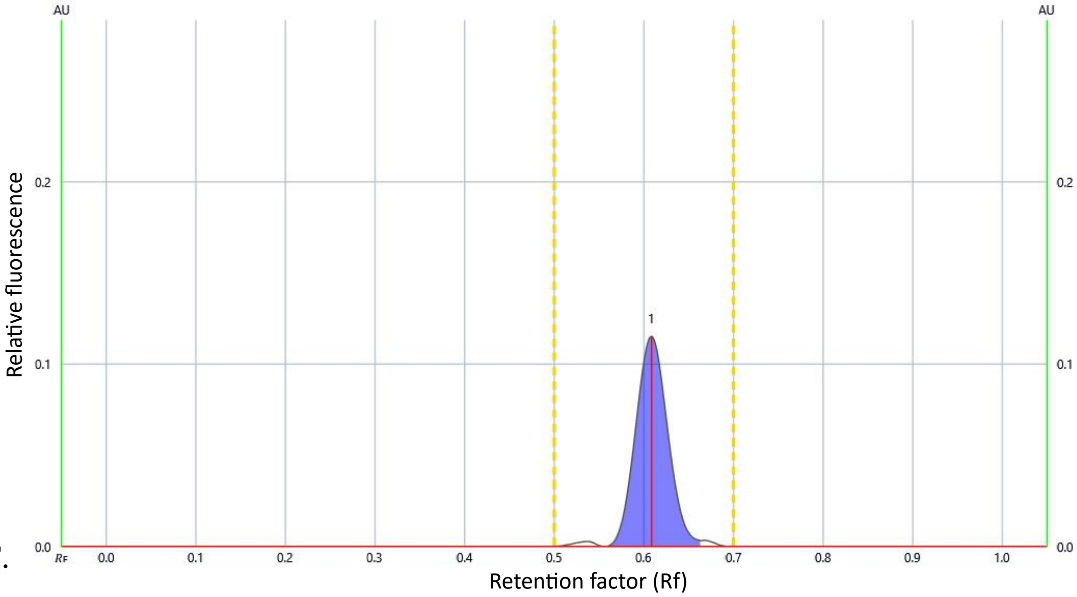


F.

**Figure S5 (continued).** Fluorescence densitograms for the determination of scopoletin (1) obtained at an excitation wavelength of 302 nm and by reading the fluorescence intensity with a K400 detection filter after consecutive development of the chromatography plate with mobile phases 6 (chloroform: ethyl acetate: formic acid (60:30:10 v/v/v)) and 7 (chloroform: ethyl acetate (60:40 v/v)). E. Densitogram obtained with the application of 100 µL of FE-20 test solution (10 mg/mL). F. Densitogram obtained with the application of 15 µL of SE-20 test solution (10 mg/mL).

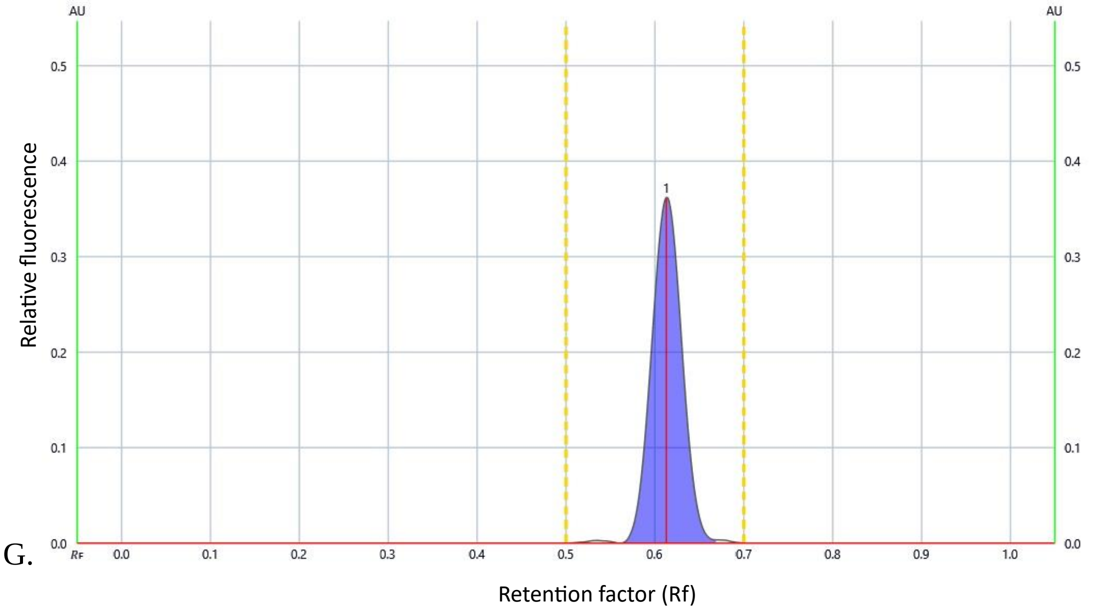


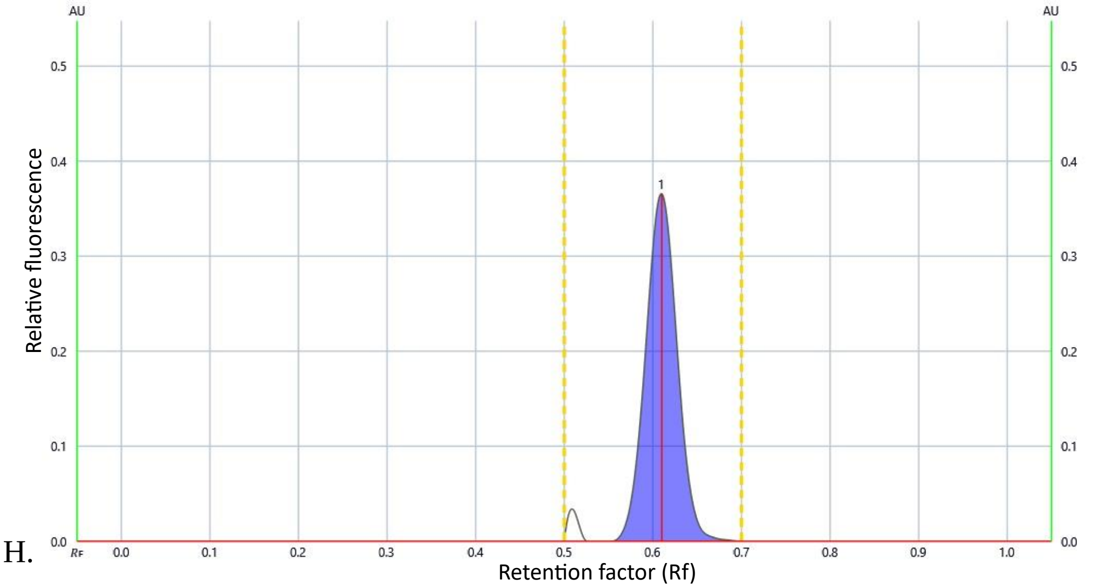


**Figure S5 (continued).** Fluorescence densitograms for the determination of scopoletin (1) obtained at an excitation wavelength of 302 nm and by reading the fluorescence intensity with a K400 detection filter after consecutive development of the chromatography plate with mobile phases 6 (chloroform: ethyl acetate: formic acid (60:30:10 v/v/v)) and 7 (chloroform: ethyl acetate (60:40 v/v)). G. Densitogram obtained with the application of 8 µL of RE-20 test solution (10 mg/mL). H. Densitogram obtained with the application of 25 µL of FL-21 test solution (10 mg/mL).

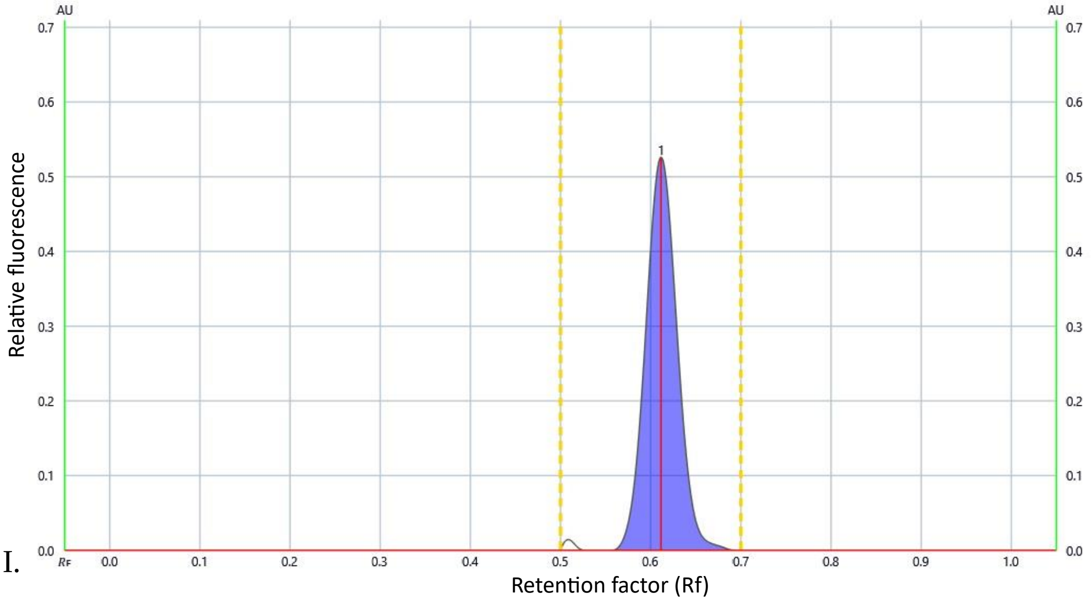


I.

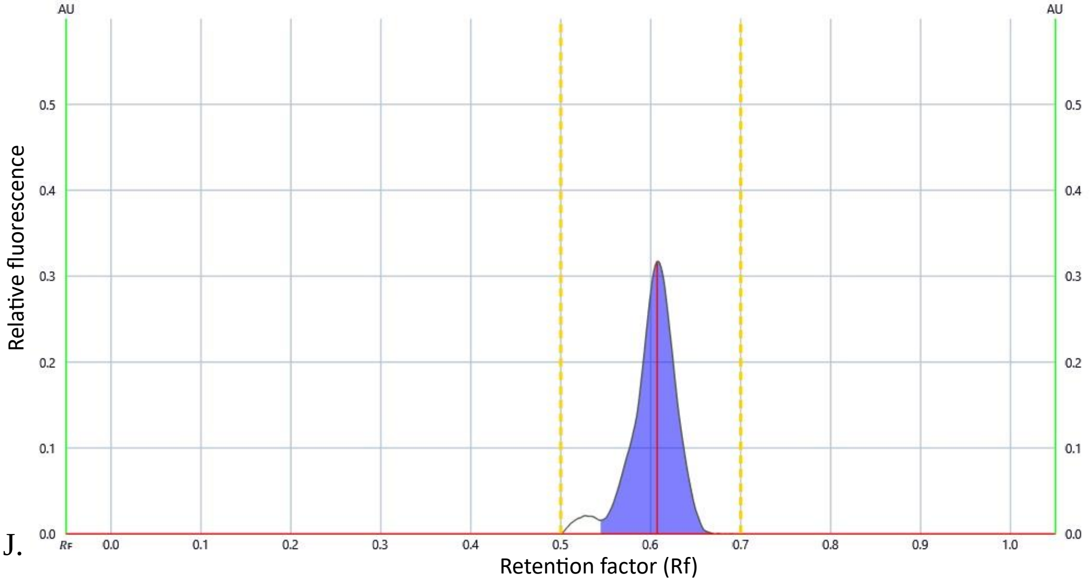


J.

**Figure S5 (continued).** Fluorescence densitograms for the determination of scopoletin (1) obtained at an excitation wavelength of 302 nm and by reading the fluorescence intensity emitted with a K400 detection filter after consecutive development of the chromatography plate with mobile phases 6 (chloroform: ethyl acetate: formic acid (60:30:10 v/v/v)) and 7 (chloroform: ethyl acetate (60:40 v/v)). I. Densitogram obtained with the application of 20 µL of OL-21 test solution (10 mg/mL). J. Densitogram obtained with the application of 100 µL of SL-21 test solution (10 mg/mL).

## 4. *In vitro* cytotoxicity evaluation of the extracts:

The *in vitro* cytotoxicity of the extracts was evaluated according to the method described by Gómez-García et al. with modifications. Medium and incubation conditions were the same as described in section 4.9.3 of the main text. Vero cells (ATCC CCL-81) were cultured in sterile 25 mL cell culture flask until 90 % of confluency and tripsinized. Then, they were resuspended with culture media to a cell density of 250 000 cells/mL. From this suspension, 100 µL aliquots were transferred to the wells of a flat bottom sterile black microplates. The plate was incubated for 22 hours to promote cell adherence to well bottoms. After this period, the cells were washed with PBS and 100 µL of extract (100 µg/mL) was added to each well. As a control of cell viability reduction, different wells were treated with 100 µL of a DMSO at concentration of 5 % v/v in culture media. Wells were reserved as 100 % cell viability control (wells with cells non treated with extracts, quercetin or DMSO 5 % v/v). For these wells 100 µL of culture media was added instead of extract, quercetin or DMSO solutions. The plate was incubated for 22 hours. After incubation of cells with the extracts, the wells were washed with PBS and 100 µL of 44 µM resazurin in phenol red free media was added. Then, the plate was incubated for 2 hours, and the fluorescence of each well was measured at excitation and emission wavelengths of 540 nm and 590 nm, respectively. The percentage of cell viability (% CV) was determined using the following equation:

$$\% CV = \frac{FE}{FC} \times 100\% \quad (1)$$

where FE denotes the mean fluorescence of wells exposed to evaluated substances (extracts, quercetin or DMSO 5% v/v) and FC is the mean fluorescence of wells designated as 100 % cell viability control. The assay was repeated in triplicate and the results are reported as average % CV with the corresponding standard error.

**Reference:** Gómez-García, M.; Puente, H.; Argüello, H.; Mencía-Ares, Ó.; Rubio, P.; Carvajal, A. In Vitro Assessment of Antiviral Effect of Natural Compounds on Porcine Epidemic Diarrhea Coronavirus. *Front. Vet. Sci.* **2021**, 8, doi:10.3389/fvets.2021.652000.

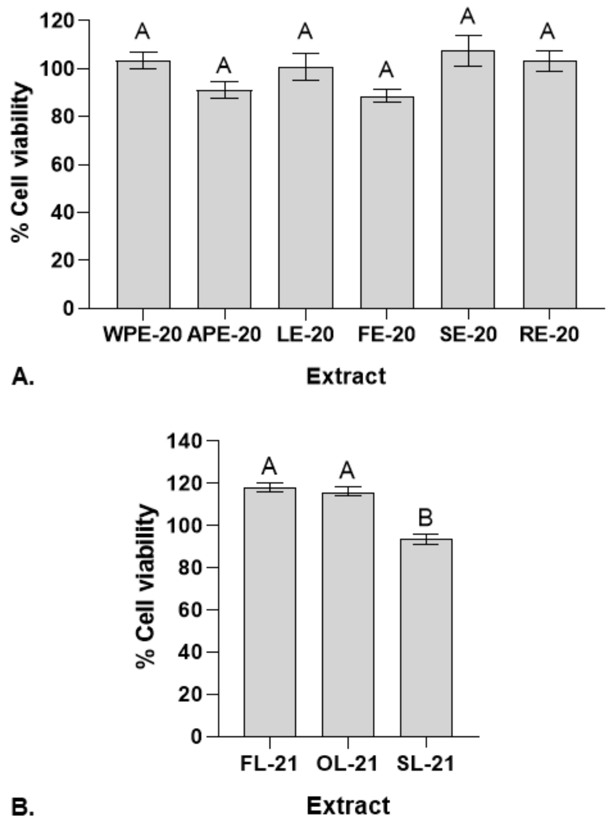


**Figure S6.** *In vitro* cytotoxicity evaluation of extracts on Vero cells (ATCC CCL-81). A. Cell viability results obtained with 100 µg/mL of extracts produced from different parts of *I. hawkeri*. B. Cell viability results obtained with 100 µg/mL of extracts produce from *I. hawkeri* leaves dried by different techniques. Cell viability percentage of cells treated with quercetin 24 µg/mL and dimethyl sulfoxide (DMSO) 5 % v/v were 95.32 ± 1.05% and 49.16 ± 1.05%, respectively. Error bars indicate standard deviation. Different letters above each bar indicates significant differences ($p$ < 0.05). WPE-20: Whole plant extract from material collected in 2020; APE-20: Aerial part extract from material collected in 2020; LE-20: Leaf extract from material collected in 2020; FE-20: Flower extract from material collected in 2020; SE-20: Stem extract from material collected in 2020; RE-20: Root extract from material collected in 2020; FL-21: Freeze-dried leaf extract from material collected in 2021; OL-21: Oven-dried leaf extract from material collected in 2021; SL-21: Shade-dried leaf extract from material collected in 2021.

**5. Dose response tendencies of the extracts in the intracellular reactive oxygen species production inhibition assay (IROS):**

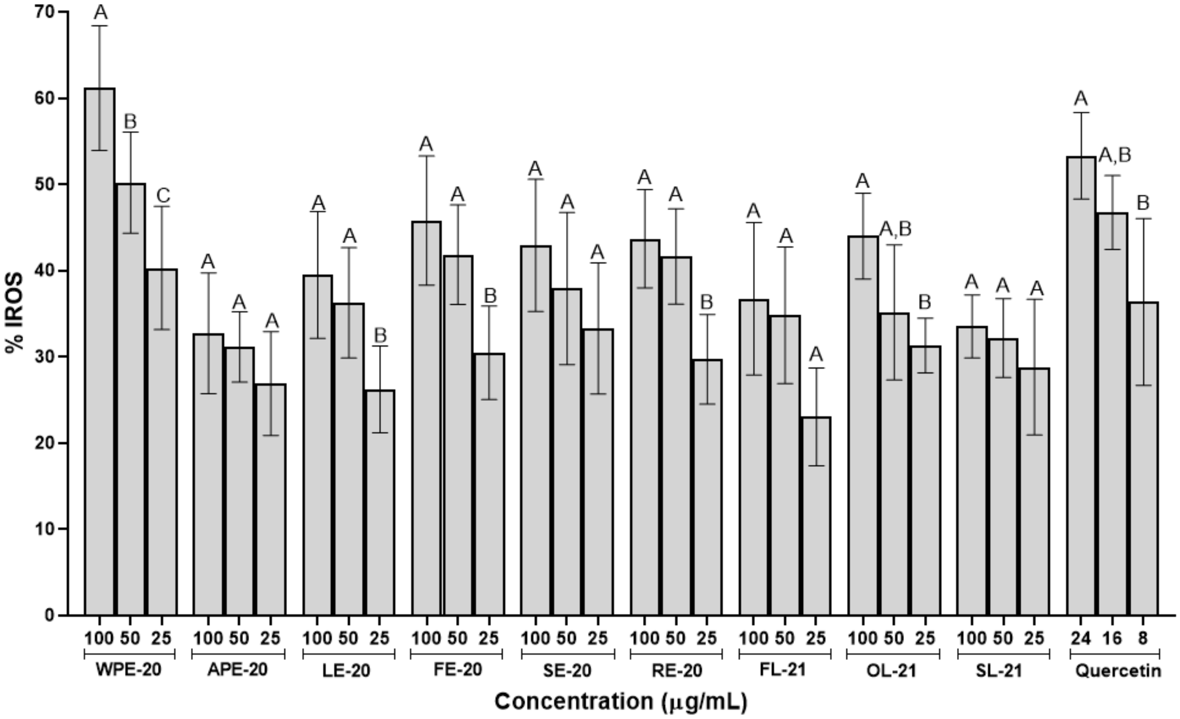


**Figure S7.** Dose response trends observed with the extracts and quercetin in the intracellular ROS production inhibition assay (IROS) on Vero cells (ATCC CCL-81). The error bars correspond to the standard deviation. Different letters above the bars of the different concentration levels for each extract indicate statistically significant differences ($p < 0.05$). WPE-20: Whole plant extract from material collected in 2020; APE-20: Aerial part extract from material collected in 2020; LE-20: Leaf extract from material collected in 2020; FE-20: Flower extract from material collected in 2020; SE-20: Stem extract from material collected in 2020; RE-20: Root extract from material collected in 2020; FL-21: Freeze-dried leaf extract from material collected in 2021; OL-21: Oven-dried leaf extract from material collected in 2021; SL-21: Shade-dried leaf extract from material collected in 2021.
